# Recent Advances in Nanozyme-Mediated Strategies for Pathogen Detection and Control

**DOI:** 10.3390/ijms241713342

**Published:** 2023-08-28

**Authors:** Tianyi Ma, Kunlun Huang, Nan Cheng

**Affiliations:** 1 Beijing Laboratory for Food Quality and Safety, College of Food Science and Nutritional Engineering, China Agricultural University, Beijing 100083, China; tianyima@cau.edu.cn (T.M.); hkl009@163.com (K.H.); 2 Key Laboratory of Safety Assessment of Genetically Modified Organism (Food Safety), Ministry of Agriculture, Beijing 100083, China

**Keywords:** nanozyme, pathogens, food safety, detection, antibacterial, therapy

## Abstract

Pathogen detection and control have long presented formidable challenges in the domains of medicine and public health. This review paper underscores the potential of nanozymes as emerging bio-mimetic enzymes that hold promise in effectively tackling these challenges. The key features and advantages of nanozymes are introduced, encompassing their comparable catalytic activity to natural enzymes, enhanced stability and reliability, cost effectiveness, and straightforward preparation methods. Subsequently, the paper delves into the detailed utilization of nanozymes for pathogen detection. This includes their application as biosensors, facilitating rapid and sensitive identification of diverse pathogens, including bacteria, viruses, and plasmodium. Furthermore, the paper explores strategies employing nanozymes for pathogen control, such as the regulation of reactive oxygen species (ROS), HOBr/Cl regulation, and clearance of extracellular DNA to impede pathogen growth and transmission. The review underscores the vast potential of nanozymes in pathogen detection and control through numerous specific examples and case studies. The authors highlight the efficiency, rapidity, and specificity of pathogen detection achieved with nanozymes, employing various strategies. They also demonstrate the feasibility of nanozymes in hindering pathogen growth and transmission. These innovative approaches employing nanozymes are projected to provide novel options for early disease diagnoses, treatment, and prevention. Through a comprehensive discourse on the characteristics and advantages of nanozymes, as well as diverse application approaches, this paper serves as a crucial reference and guide for further research and development in nanozyme technology. The expectation is that such advancements will significantly contribute to enhancing disease control measures and improving public health outcomes.

## 1. Introduction

Bacterial infection remains a significant global security threat to human health [1,2,3]. Antibiotics derived from natural products or chemically modified natural compounds have become the most commonly used approach for treating bacterial infections [4,5]. Antibiotics have played a significant role in the prevention, containment, and treatment of infectious diseases in animals [6]. The extensive utilization of antibiotics has also contributed to the significant issue of bacterial resistance [7,8,9]. More significantly, the proliferation of bacterial resistance poses a grave menace to global public health [9]. However, conventional methods for pathogen detection, such as a cell culture [10,11,12], polymerase chain reaction (PCR) [13,14,15], enzyme-linked immunosorbent assay (ELISA) [16,17,18], and other techniques, are time-consuming, technically demanding, and require specialized equipment. Moreover, they cannot achieve rapid and efficient detection in non-laboratory settings, which hinders the prompt discovery and effective control of pathogens [19]. Hence, it poses a significant challenge to explore novel strategies for achieving efficient detection and control of pathogens.

To tackle this challenge, significant efforts have been directed toward the development of efficient, streamlined, and rapid pathogen detection methods as well as alternative broad-spectrum antibacterial agents or strategies. For instance, certain detection platforms and antibacterial materials that utilize nanomaterials, such as continuous cascade nanozyme biosensors [20], cascade methods based on a natural self-assembled nanozyme [21], a nanozyme-mediated CRISPR-Cas 12a system [22], photothermal therapy (PTT) [23], photodynamic therapy (PDT), and photocatalytic therapy (PCT) [24], are emerging [25]. Recently, nanozyme-based sensing platforms have demonstrated successful applications in the diagnosis of bacteria, viruses, and plasmodium parasites [22,26,27]. In addition, the natural peroxidase (POD) and oxidase (OXD) present in living organisms can catalyze a range of substrates to generate numerous reactive oxygen species (ROS), which are effective against bacterial invasion [28]. Therefore, nanozymes are anticipated to serve as a viable alternative to antibiotics owing to their exceptional catalytic activity, remarkable substrate selectivity, and gentle action conditions [23]. However, the utilization of natural enzymes in pathogen diagnoses and control is greatly limited due to their high production costs, complex conditions, low stability of catalytic activity, and difficulty in storage [29,30]. In 2004, Scrimin and his colleagues employed triazabicyclo-alkane-functionalized gold nanoparticles as a catalyst for the dephosphorylation reaction [31]. In 2007, Yan’s team initially reported that Fe_3_O_4_ magnetic nanoparticles could serve as a peroxidase mimic similar to horseradish peroxidase (HRP) [32]. Recently, nanozymes have emerged as a research hotspot due to their promising features of a low cost, a high stability, facile production, tunable catalytic activity, and convenient storage [33,34,35,36]. To date, nanozymes utilized for antibacterial and pathogen detection primarily consist of metal-based compounds, carbon-based nanomaterials, transition metal dichalcogenides/peroxides/oxides, single-atom nanozymes (SAzymes), and metal–organic frameworks (MOFs)-based compounds that have been extensively developed [31,37,38].

Previously, several comprehensive reviews have provided systematic overviews of the applications of nanozymes in pathogen detection and control. Nguyen et al. extensively discuss the sensing mechanisms, device structures, analytical characteristics, and relevant applications of nanomaterials in colorimetric paper-based biosensors for accurate pathogen detection [39]. Songca et al. investigate the enhancement of the mechanisms, design, and structural role of nanozyme nanoconjugates in the detection and identification of viral, bacterial, and fungal pathogens [40]. Additionally, Sun et al. present a comprehensive overview of various nanozymes and their applications in pathogen detection [31]. However, there is currently no exhaustive review or exposition on the antibacterial and detection mechanisms employed with nanozymes.

**Figure 1:**
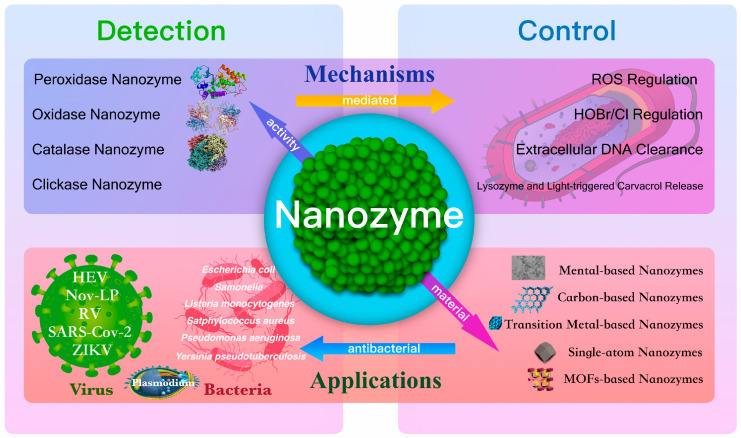
A concise overview of nanozymes regarding their mechanisms, mimetic categories, targets, and classifications.

The aim of this review is to provide a comprehensive review on the recent advancements in various nanozymes for combating bacterial infections and detecting pathogens. Additionally, it aims to elucidate the physicochemical properties and mechanism of action of nanozymes in antibacterial detection and pathogenic bacteria identification. This study differs from previous research endeavors. By categorizing and summarizing the antibacterial and detection mechanisms employed with these nanozymes, we describe their scope, efficacy, as well as factors influencing their performance in controlling pathogenic microorganisms (Figure 1). Furthermore, we analyze the main challenges faced by nanozymes in pathogen detection and control, while also exploring their future potential. Our intention is that this review will inspire readers with novel insights and technologies for pathogen detection and management, fostering a more comprehensive understanding of antimicrobial strategies based on nanomaterials.

## 2. Nanozyme-Mediated Detection Mechanisms

Rapid and efficient pathogen detection is a crucial component of disease prevention and control. Currently, commonly used immunoassays for detection, such as high-performance liquid chromatography (HPLC), gas chromatography (GC), mass spectrometry (MS), gas chromatography/mass spectrometry (GC-MS), liquid chromatography/mass spectrometry (LC-MS), and ELISA, exhibit exceptional specificity and accuracy [41]. However, these methodologies often require complex pre-processing procedures, advanced instrumentation, and skilled technical personnel for implementation, resulting in extended detection timelines that present challenges in meeting the demands of real-time pathogen detection [42]. Enzyme-mimetic nanomaterials, also known as nanozymes, are highly promising tools for enhancing quality assessment and ensuring safety in the agri-food industry due to their ability to mimic enzymes and possess nanoscale features. Their robust detection capabilities and potential for future-oriented applications make them particularly attractive for utilization in this sector [43]. In contrast to their natural enzyme counterparts, nanozymes possess several advantageous features including cost effectiveness [44], facile scalability for mass production [45], resilience towards harsh environmental conditions [46], prolonged storage capability [47], exceptional stability, and the ability to customize their activities as per specific requirements [45]. Nanozymes have demonstrated tremendous potential in surpassing the limitations of conventional enzymes within the field of analyses. Consequently, it is of significant importance to categorize the underlying principles of nanozymes within the context of pathogen detection. The catalytic activity of a nanozyme relies on the surface electron transfer process [48]. Researchers employed electron spin resonance (ESR) [49], density functional theory (DFT) [50], X-ray photoelectron spectroscopy (XPS) [51], and ultraviolet-visible spectrophotometry (UV) [52] to investigate the catalytic reaction mechanism of a nanozyme while employing an enzyme steady-state kinetic analysis to examine its catalytic activity. Nanozymes can be classified based on their catalytic types, which are determined by their distinct catalytic mechanisms [53]. This discourse primarily covers five distinct categories of nanozymes, including peroxidase-mimicking, oxidase-mimicking, catalase-like, glucose-oxidase-mimicking, and clickase nanozymes. All of these demonstrate the ability to detect key analytes relevant to the agri-food industry.

### 2.1. Peroxidase Nanozyme

**Figure 2:**
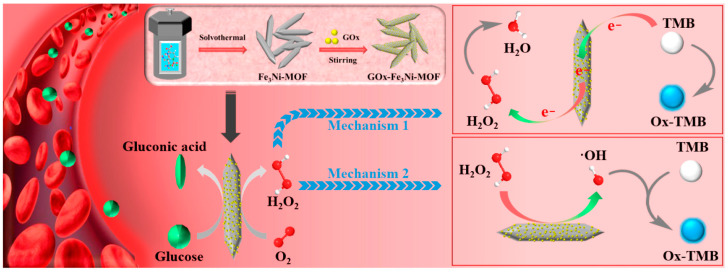
Schematic diagram of the synthesis, mechanism, and application of Fe_3_Ni-MOF/GOx (reproduced from Mu et al., 2022) [59].

POD mimics, a widely utilized category of nanozymes in detection applications, play an indispensable role in facilitating the catalytic conversion of a substrate into its oxidized form. This enzymatic process generates a variety of signals, including colorimetric manifestations (2,2′-azino-bis(3-ethylbenzothiazoline-6-sulfonic acid) (ABTS), 3,3′,5,5′-tetramethylbenzidine (TMB), and o-phenylenediamine (OPD)), fluorescent manifestations such as Ampliflu Red, and chemiluminescent manifestations like luminol. These signals become distinguishable in the presence of hydrogen peroxide (H_2_O_2_) as a cosubstrate. The catalytic kinetics of POD-like nanozymes are known to exhibit variations in response to appropriate concentration ranges of standard substrates and H_2_O_2_ cosubstrates, resulting in the generation of divergent quantities of catalytic oxidation products. As a result, various analytical techniques have been developed for a food analysis utilizing POD nanozymes to measure the cosubstrate H_2_O_2_ [54,55,56,57]. Simultaneously, certain substances exhibit selective reactivity towards H_2_O_2_, enabling signal amplification facilitated by the catalytic activity of nanozymes. The concentration of the substrate can be determined by monitoring the consumption of H_2_O_2_, thus achieving quantitative detection objectives [57,58] (Figure 2). Furthermore, complex reactions can be achieved through the synergistic interplay of multiple nanozymes. In order to construct an artificial cascade detection system, the researchers integrated nanozymes with hydrogen peroxide generation ability and POD nanozymes [55,58]. Similarly, it is possible to devise detection systems that incorporate cascade reactions, in which two or more nanozymes are collaboratively utilized autonomously without the involvement of enzymes [43]. In summary, this description highlights the application of POD mimics as nanozymes in detection scenarios, demonstrating their versatility in generating diverse signals and potential for quantification in a food analysis. Furthermore, the synergistic actions of nanozymes present opportunities for intricate detection systems, offering innovative approaches for analytical purposes.

### 2.2. Oxidase Nanozyme

Furthermore, nanozyme-based detection platforms can be utilized through the consumption of catalytic oxidation products (ox-substrate), providing access not only to peroxidase-like nanozymes but also oxidase nanozymes. Zhou and colleagues demonstrated the application of MnO_2_ nanosheets with oxidase-mimicking activity in catalyzing the oxidation of TMB to TMB^2+^. Subsequently, the gold nanorods (Au NRs) underwent rapid etching, resulting in a noticeable blue shift of their longitudinal local surface plasmon resonance peaks and polychromatic changes. In the presence of *E. coli*, a cascade reaction was initiated via β-galactosidase hydrolysis of p-aminophenol β-D-galactopyranoside (PAPG), resulting in the generation of p-aminophenol (PAP). Subsequently, PAP mediated the reduction of MnO_2_ nanosheets, leading to the disruption of their oxidase-mimicking activity and subsequently affecting TMB^2+^ generation. Consequently, the sensing system is capable of easily detecting varying concentrations of *E. coli* through distinct color changes exhibited [60]. The integration of MnO_2_ nanosheets and Au NRs not only facilitated the detection of *E. coli* but also enabled the observation of visual color changes, thereby providing a promising strategy for achieving sensitive and selective detection in the presence of the bacteria.

### 2.3. Catalase-like Nanozyme

**Figure 3:**
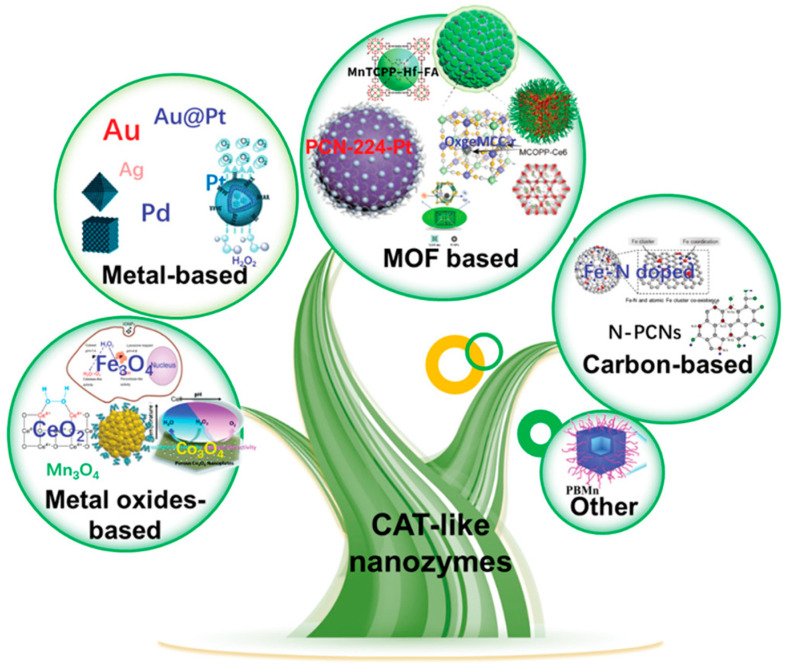
Illustration of the classification of CAT-like nanozymes based on different nanomaterials (reproduced from Deting et al., 2022) [62].

Catalase (CAT) is a vital oxidoreductase in the human body, which plays a significant role in catalyzing the elimination of ROS, safeguarding cell membrane integrity, and inhibiting tumor cell growth [61]. CAT-like nanozymes are a class of nanomaterials that possess inherent catalase activity, enabling them to efficiently facilitate the decomposition of H_2_O_2_ into water (H_2_O) and oxygen (O_2_) molecules [62]. Nano-enzymes with CAT activity based on different nanomaterials include metals, metal oxides, metal–organic frameworks (MOFs), carbon-based nanomaterials, metal sulfides, and Prussian blue (PB) (Figure 3) [62]. Zhou’s research team successfully synthesized trimetallic AuPtCo nanopolyhedrons that exhibit peroxidase and catalase-like catalytic activities. In an alkaline environment, the presence of ABEI and H_2_O_2_ leads to their conversion into an ABEI anion (ABEI^−^) and HO^2−^, respectively. Furthermore, the H_2_O_2_ is decomposed by AuPtCo@Cys into HO^•^ and O_2_^•−^ through catalysis. Subsequently, ABEI- reacts with the generated HO^2−^ and HO^•^ to form ABEI^•−^ and O_2_^•−^, respectively. Notably, O_2_^•−^ continuously reacts with HO^•^ to produce a plentiful supply of O_2_. Ultimately, ABEI^•−^ reacts with both O_2_^•−^ and O_2_, resulting in the formation of the excited-state oxidation product of ABEI (ABEI-ox*), which subsequently returns to its ground state accompanied by light emission [63]. The developed approach successfully achieved sensitive detection of H_2_O_2_ and lipoprotein-associated phospholipase A2 (Lp-PLA2) in actual samples, thereby demonstrating its potential to significantly broaden the application scope in the field of rapid pathogen detection. These findings offer promising prospects for the expansion of pathogen rapid detection applications, emphasizing the potential utility of the synthesized nanopolyhedrons in this specific field.

### 2.4. Clickase Nanozyme

**Figure 4:**
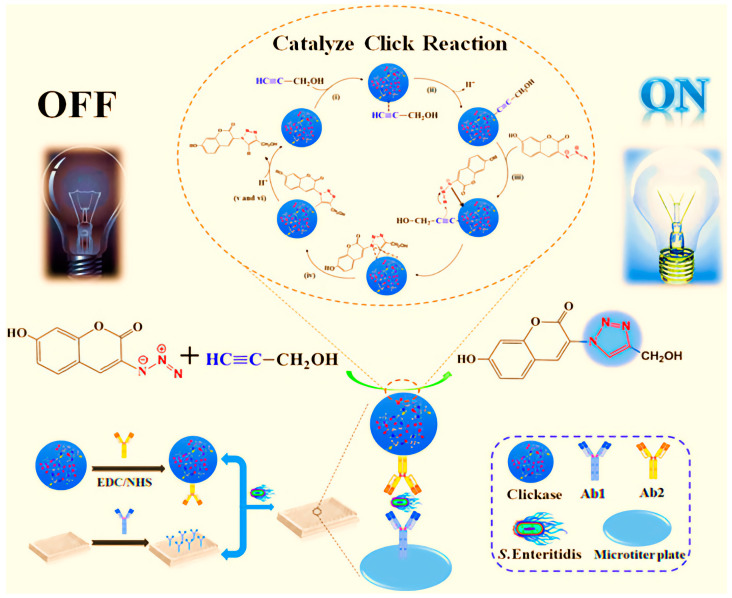
The stability and enzyme-like catalytic activity in the CuAAC reaction of the CCN clickase, and the catalytic mechanism of the CCN-clickase-mediated CuAAC reaction between 3-azide-7hydroxycoumarin and propargyl alcohol (reproduced from Zhang et al., 2021) [64].

Clickase has demonstrated its efficacy in streamlining reactions. Currently, a Cu(I)-based nanocatalyst has been developed for the detection and analysis of foodborne pathogens, which is rich in Cu(I) ions effectively stabilized by glutathione (GSH). Furthermore, the Cu(I)-containing nanomaterials-based clickase exhibits exceptional biocompatibility, which facilitates antibody modification due to the abundant presence of amino (-NH_2_) and carboxyl (-COOH) groups on its surface (Figure 4) [64]. Moreover, a nanoflower-like artificial clickase, known as LCN clickase, has been introduced for the portable and sensitive detection of foodborne bacterial pathogens using a click SERS immunoassay. Benefiting from its remarkable catalytic activity that promotes the Cu(I)-catalyzed azide–alkyne cycloaddition reaction, LCN clickase has successfully enabled the development of a novel click SERS immunoassay. This innovative approach integrates the clickase-mediated SERS signal modulation in the Raman-silent region, thereby effectively eliminating interferences caused by Raman reporters and biological components while minimizing complex sample matrix interference. In comparison to conventional CuAAC-based immunoassays, the established method avoids the unnecessary requirement of a dissolution process for nanocatalysts and eliminates the necessity for a reducing agent in the detection process. As a result, the detection time is considerably shortened, and the detection reliability is enhanced [65]. Fundamentally, clickase and its derivatives, such as CCN and LCN clickase, have revolutionized the field of biochemistry, particularly in foodborne pathogen detection. These advancements have significantly streamlined reaction processes, improved the reliability and sensitivity of detection methods, and substantially reduced detection time.

Due to their distinctive advantages and comparable catalytic capabilities to enzymes, nanozymes have garnered increasing attention for their applications in detection. Specifically for pathogen detection, nanozymes offer a simplified preparation and purification process compared to natural enzymes, obviating the need for intricate equipment or instruments [45]. Motivated by extensive research on nanozymes and an increasingly profound understanding of nanoscale particles, contemporary preparations of nanozymes have exhibited reduced time requirements and demonstrated efficacy in meeting diverse analytical demands [66]. Nanomaterial-based platforms have demonstrated enhanced efficacy, accelerated detection speed, and broader pathogen coverage in contemporary applications for pathogen detection.

### 2.5. Application of Nanozymes in Bacteria Detection

Given the increasing prevalence of foodborne illnesses, foodborne bacteria have emerged as a significant threat to global public health in recent years [67]. The efficient identification of pathogenic bacteria is crucial for the successful implementation of control measures. In the field of foodborne pathogen detection, biosensors incorporating nanozymes have been widely used [38].

#### 2.5.1. *Escherichia coli*

The successful implementation of effective control measures relies on the utilization of efficient, rapid, and highly sensitive detection methods specifically designed for targeting pathogenic bacteria [68]. Zhang et al. developed man-PB, a functional nanozyme, for rapid and label-free detection of *E. coli O157:H7* using a multi-readout LFIA technique. The man-PB nanoparticles showed exceptional binding affinity towards the flagella of *E. coli O157:H7* as a novel recognition agent, enabling efficient capture of the target pathogen [56]. Additionally, He et al. developed a detection strategy utilizing nanozymes in a cascade reaction. When exposed to an acidic solution, MnO_2_ nanosheets exhibited catalytic activity and converted the colorless substrate TMB into TMB^2+^ ions. Acting as an oxidant, TMB^2+^ rapidly corroded Au NRs, resulting in noticeable color changes. In our sensing methodology, β-gal derived from *E. coli* initiated the cascade reaction. β-galactosidase catalyzed the hydrolysis of the PAPG substrate, resulting in the formation of a reducing agent called PAP. PAP was found to reduce MnO_2_ nanosheets, inhibiting their oxidase activity and subsequently decreasing the production of TMB^2+^. Consequently, the etching process of Au NRs was impeded. The diverse colors resulting from the etching of Au NRs facilitated a semi-quantitative visual analysis of β-galactosidase in *Escherichia coli*. The synergistic implementation of enzyme–nanozyme cascade reactions and the unique optical properties of Au NRs significantly enhanced the detection sensitivity towards β-galactosidase in *E. coli*. Moreover, this approach offered a high-resolution and user-friendly visual method for determining the concentration of *E. coli* through direct observation with the naked eye [60]. These groundbreaking methodologies highlight the use of functional nanozymes and cascade reactions based on nanozyme technology as highly effective and sensitive means for detecting pathogenic bacteria.

#### 2.5.2. *Listeria monocytogenes*

*Listeria monocytogenes* (*L. monocytogenes*) are Gram-positive foodborne pathogens that exhibit a significant presence across diverse food sources [69]. Shi et al. proposed an innovative approach for the detection of viable *L. monocytogenes* by integrating PMA-LAMP technology with a nanozyme strip. This integrated platform allows for instrument-free, highly sensitive, and visually perceptible identification of viable *L. monocytogenes*. By modifying primers with BIO and FITC moieties, the LAMP process can generate a modified hlyA gene that specifically targets *L. monocytogenes*. Subsequently, magnetic nanozymes functionalized with biotin antibodies are used to create a probe capable of specifically recognizing and isolating *L. monocytogenes* on the strip [70]. Wu et al. have developed a magnetic resonance spectroscopy (MRS) DNA sensor utilizing an Au@Pt nanozyme for the rapid and highly sensitive detection of *L. monocytogenes* in chicken samples. In the presence of *L. monocytogenes*’ DNA, Au@Pt-probe2 nanoparticles demonstrate specific binding affinity to the target DNA region. Subsequently, the formed Au@Pt-probe_2_-target DNA conjugates are hybridized with probe 1-modified magnetic nanoparticles (MNP _180_-probe_1_). Following magnetic separation, the Au@Pt nanozyme within the conjugates catalyzes an enzymatic reaction involving H_2_O_2_, thereby facilitating the conversion of paramagnetic ions from Mn(VII) to Mn(II). The conversion mentioned above induces a corresponding alteration in the signal of transverse relaxation time (ΔT_2_), thereby facilitating the precise and sensitive detection of *L. monocytogenes* [71]. Both Shi et al.’s PMA-LAMP combined with a nanozyme strip and Wu et al.’s Au@Pt-nanozyme-mediated MRS DNA sensor represent innovative approaches for the detection of viable *L. monocytogenes*. The method proposed by Shi et al. offers an instrument-free and visually discernible qualitative detection mechanism through the integration of PMA-LAMP and a nanozyme strip. Conversely, the approach proposed by Wu et al. utilizes an Au@Pt-nanozyme-mediated MRS DNA sensor, which enables rapid and sensitive detection through monitoring changes in the transverse relaxation time (ΔT_2_) signal. These significant advancements contribute to the field of food safety by providing efficient and reliable detection methods for L. monocytogenes across various food sources.

#### 2.5.3. *Salmonella*

The World Health Organization (WHO) has made an estimation highlighting that approximately 10% of the global population experiences foodborne illnesses annually, leading to the loss of 33 million healthy life years on a yearly basis [72]. *Salmonella* is one of the four major global etiological factors contributing to the occurrence of diarrheal diseases [73,74]. Guo et al. have proposed a microfluidic immunosensor as an innovative approach for the detection of *Salmonella*. The immunosensor employs Fe-MOF/PtNPs as a signal amplifier, a smartphone-based thermal sensor as a signal detector, and a two-layer microfluidic chip as a research platform. In this system, magnetic nanoparticles (MNPs) coated with capture antibodies (CAbs, indicated in green), *Salmonella*, and detection antibodies (DAbs, marked in purple) modified with Fe-MOF/PtNPs are simultaneously introduced into the microfluidic chip. Efficient mixing is facilitated by a three-dimensional chaotic micromixer followed by adequate incubation in the dedicated channel to form sandwich complexes consisting of MNPs–*Salmonella*–Fe-MOF/PtNPs. Subsequently, a magnet is utilized to capture these complexes within a separation chamber. In order to initiate a reaction, H_2_O_2_ is injected and decomposed by the nanozyme present on the sandwich complexes, resulting in the production of O_2_ within the sealed chamber. The rise in pressure within the separation chamber propels preloaded H_2_O into the heating chamber containing calcium oxide (CaO) powder, thereby inducing heat generation. The quantity of *Salmonella* is ascertained by gauging the resultant thermal output using a smartphone-based thermal sensor [75]. Wang et al. have reported the synthesis of Fe_3_O_4_@Cu (His-Fe_3_O_4_@Cu) magnetic nanozymes, which demonstrate remarkable peroxidase-like activity and strong superparamagnetism. These nanozymes have been employed to establish a straightforward and highly sensitive colorimetric method for the detection of *Salmonella typhimurium* (*S. typhimurium*). In their study, an Apt-His-Fe_3_O_4_@Cu complex modified with aptamer 1 and effectively blocked non-specific binding sites using whey protein isolate (WPI) was developed as a capture agent for selective enrichment of *S. typhimurium* in food samples. Subsequently, biotin-aptamer 2 is immobilized onto a microplate via the specific binding between avidin and biotin, serving as the capture probe for selective recognition of *S. typhimurium*, resulting in the formation of microplate/*S. typhimurium*/His-Fe_3_O_4_@Cu “sandwich” complexes. After eliminating any unbound Apt-His-Fe_3_O_4_@Cu, a hydrochloric acid (HCl) etching solution is introduced to restore the peroxidase-like activity of His-Fe_3_O_4_@Cu. Ultimately, the detection signal produced in the presence of a TMB-H_2_O_2_ solution can be visually perceived by naked eyes or quantitatively measured using a SpectraMaxi3x Reader [76]. These methodologies free Salmonella detection from the requirement of bulky instrumentation, allowing for sensitive, rapid, accurate, and efficient detection. By utilizing microfluidic systems’ integration, signal amplification techniques, and nanozyme technology, these investigations have established sensitive, expeditious, and efficient approaches to discerning and quantifying Salmonella in food specimens. The aforementioned advancements thus make a significant contribution to the enhancement of food safety standards and the safeguarding of public health.

#### 2.5.4. *Staphylococcus aureus*

*Staphylococcus aureus* (*S. aureus*) is acknowledged as a significant foodborne pathogen capable of producing enterotoxins, which are implicated in the etiology of various severe and multifaceted ailments. These encompass localized suppurative infections, pneumonia, pseudomembranous colitis, pericarditis, sepsis, and diverse systemic infections [77]. Based on available data, *Staphylococcus aureus* has been identified as one of the prominent pathogens contributing to acquired foodborne illnesses and ranks among the top five. In the United States alone, this bacterium is estimated to be responsible for approximately one million cases of such illnesses annually [78]. Consequently, the scientific community has placed significant emphasis on developing robust and highly sensitive detection methods for *Staphylococcus aureus* [79]. The conventional method for detecting *Staphylococcus aureus* involves a process of selective enrichment, followed by an enumeration of colonies based on morphological characteristics. Additional evaluation may involve staining techniques or enzymatic assays for identification purposes. Regrettably, these procedures typically require a time frame of 2–4 days to produce informative results, making them unsuitable for urgent situations such as public health emergencies or cases of foodborne illness [80]. Numerous highly sensitive detection methods for *Staphylococcus aureus* have been developed by researchers, utilizing the PCR technique [81]. Furthermore, the application of ELISA has been investigated as a viable alternative for detecting *Staphylococcus aureus* with favorable sensitivity levels [61,82]. Despite the inherent advantages of these detection strategies, such as their ability to achieve low detection limits and high specificity, their widespread implementation is hindered by certain challenges. Specifically, the need for expensive instrumentation and a demand for well-trained personnel act as barriers to their broader application [81].

Aptamers are functional single-stranded oligonucleotide sequences, possessing the unique ability to selectively bind to targets with remarkable affinity. These aptamers are obtained through an in vitro process known as the Systematic Evolution of Ligands by Exponential Enrichment (SELEX) method [83]. Aptamers, akin to antibodies, demonstrate remarkable affinity and specificity toward a wide range of targets, including proteins, small molecules, ions, and even cells [84]. In a recent study conducted by Zhu et al., it was discovered that oligonucleotide sequences, specifically aptamers, possess the ability to adsorb onto the surface of octahedral Mn_3_O_4_ nanoparticles (NPs) and modulate their oxidase-mimicking activity. This intriguing phenomenon has facilitated the development of an advanced label-free and highly sensitive colorimetric aptasensor for *Staphylococcus aureus* detection [85]. The integration of oligonucleotides has facilitated the modulation of a Mn_3_O_4_ nanoparticle’s oxidase-mimicking activity, resulting in a cutting-edge colorimetric aptasensor for the highly sensitive and selective detection of *Staphylococcus aureus* in food samples [85]. The interaction between octahedral Mn_3_O_4_ NPs and the chromogenic substrate TMB results in a hindered binding effect. As a consequence, the oxidase-mimicking activity of the catalytic system is significantly impeded, leading to electron loss from a fraction of TMB molecules and subsequent formation of oxidized TMB (oxTMB), which is observed as blue or green products. In the presence of *S. aureus*, the SA31 aptamer sequences exhibit a preferential binding affinity towards their corresponding target bacteria, resulting in the formation of stable complexes. Consequently, this alleviates the inhibitory effect on the oxidase-mimicking activity of octahedral Mn_3_O_4_ NPs. The induction of a visible color change is observed in the developed colorimetric aptasensor, which exhibits exceptional sensitivity with a low detection limit of 3 colony-forming units (CFU) per milliliter (mL). Additionally, it demonstrates excellent selectivity for detecting *S. aureus*.

To overcome the limitations of conventional ELISA techniques, such as colorimetric reactions, substantial sample volume requirements (approximately 200 μL), extended processing time (1–2 h), and the need for bulky instrumentation to interpret test results [86], the utilization of paper-based enzyme-linked immunosorbent assay (P-ELISA) tests has emerged as a promising solution to address these challenges, particularly in point-of-care testing (POCT) settings [87]. P-ELISA tests offer additional advantages, such as cost effectiveness and ease of use. Moreover, the test results can be visually discerned without relying on specialized detection instruments. Currently, there is a convergence between the P-ELISA concept and Pd@Pt nanoparticles, leading to the development of a diagnostic test known as the “nanozyme-immobilized paper-based analytical device” (nPAD) [88]. To improve the stability of the P-ELISA system, the researchers developed a 3D-printed sample holder that facilitates secure alignment and optimal contact between the multi-layered paper components comprising the test platform. Subsequently, the integration led to the development of an nPAD that enables rapid and precise test results with a turnaround time of approximately 30 min. Additionally, the nPAD demonstrated exceptional sensitivity, achieving a low limit of detection (LOD) as minimal as 9.56 ng/mL when used for quantifying protein A, a specific biomarker utilized in *Staphylococcus aureus* detection.

Concurrently, the implementation of a nanozyme-based enzyme-linked immunosorbent assay (N-ELISA) demonstrates significant improvements in achieving lower LOD compared to conventional ELISA techniques [89,90]. Wang’s research team has developed a systematic and portable application of a Pd@Pt nanozyme for the detection of Staphylococcus aureus using N-ELISA. They conducted a comparative study investigating three different immunoassay methods to explore a more sensitive detection strategy. Wang’s research team has systematically and portably applied Pd@Pt nanozymes in detecting *Staphylococcus aureus* through N-ELISA. A comparative study was conducted on three immunoassay methods to explore a more sensitive detection strategy. Type I utilized the conventional ELISA method, while Type II employed a modification that replaced the HRP-conjugated goat anti-mouse IgG (HRP-IgG) with a Pd@Pt-nanozyme-labeled secondary antibody (Pd@Pt-IgG). Furthermore, Type III introduced an enhanced bioprobe (Pd@Pt-HRP-IgG) by coupling HRP-IgG with Pd@Pt nanozymes to leverage their surface properties and achieve improved catalytic performance. Through his strategic integration, the LOD was significantly improved from 1 × 10^5^ to 1 × 10^3^ colony-forming units per milliliter (CFU/mL). As a result, this innovative approach has the potential to meet more stringent detection regulations using traditionally simple methods such as ELISA [79].

**Figure 5:**
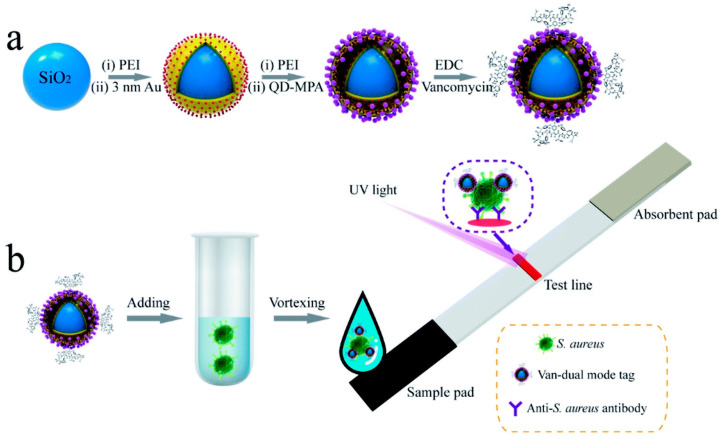
The diagram presented in (**a**) illustrates the synthesis process of the vancomycin-modified dual-signal tag, while (**b**) demonstrates the mechanisms employed for the rapid detection of Staphylococcus aureus using a dual-signal tag-based lateral flow assay (LFA) strip (reproduced from Wang et al. (2021) [91] under the Creative Commons Attribution-NonCommercial 3.0 Unported Licence).

Vancomycin demonstrates high affinity and binds to the D-Ala-D-Ala moiety present in the peptidoglycan structure of bacterial cell walls through robust hydrogen bonding interactions and other related mechanisms (Figure 5) [91]. The literature has documented the use of vancomycin-functionalized nanomaterials for selectively capturing pathogenic bacteria in complex sample solutions [92]. In this study, researchers utilized vancomycin-modified magnetic nanoparticles (Fe_3_O_4_@Au-Van MNPs) to efficiently capture and isolate bacteria in complex environmental conditions. Additionally, by synergistically utilizing the specific binding capabilities of aptamers and the oxidase-like activity of octahedral Mn_3_O_4_ NPs, a novel magnetic-assisted colorimetric biosensor was developed. This biosensor exhibited exceptional sensitivity and selectivity in detecting pathogens in complex samples, without the need for laborious pretreatment steps. Notably, the Fe_3_O_4_@Au-Van MNPs and Mn_3_O_4_ NPs utilized in the colorimetric biosensor maintained their high reactivity even after multiple cycles of use, presenting a reliable and cost-effective solution for industrial applications in pathogen detection. A comparative analysis with similar colorimetric approaches demonstrated several advantages of the constructed biosensor. These include a lower limit of detection (2 CFU·mL^−1^), an extended linear range (1 × 10^6^ CFU·mL^−1^), reduced interference from matrix effects, and enhanced specificity [93]. The advancement of immunoassay techniques, specifically P-ELISA and N-ELISA, in conjunction with the integration of nanomaterials like Pd@Pt nanoparticles, has brought about a paradigm shift in the field. These developments have resulted in significant enhancements in sensitivity, reduced sample volume requirements, shortened processing durations, cost effectiveness, and improved user friendliness. Consequently, these advancements offer significant potential to enhance diagnostic capabilities and facilitate more efficient and accessible testing across a range of applications, including disease detection and monitoring.

#### 2.5.5. *Pseudomonas aeruginosa*

*Pseudomonas aeruginosa* (*PA*) is an opportunistic, Gram-negative pathogen of significant clinical relevance due to its capacity to elicit a wide range of severe nosocomial bloodstream infections [94,95]. According to the guidelines set forth by WHO, *PA* is recognized as an indicator organism for evaluating drinking water quality. Additionally, due to its ubiquitous environmental presence, *PA* is among the most notorious pathogens responsible for water contamination in various regions worldwide, including Asia, Europe, and the United States [95,96]. The unique surface plasmon resonance and catalytic characteristics exhibited by gold nanoparticles (GNPs) have made them indispensable in the field of colorimetric detection. In this context, GNPs act as catalysts for the oxidation of TMB, leading to the formation of diamine, which subsequently undergoes conversion to diimine, an electroactive product of interest [97,98]. To exploit the electroactive properties of TMB for electrochemical sensing, researchers have developed an aptamer–nanozyme-based assay on commercially available disposable screen-printed electrodes. This integration enables the acquisition of an electrochemical readout that facilitates highly sensitive detection of *PA*. The integration of the nanozyme assay with an electrochemical platform presents significant enhancements in diagnostic sensitivity. The aptamer–nanozyme-based electrochemical approach demonstrates remarkable features, including rapidity (within 10 min), affordability, and exceptional sensitivity (detecting concentrations as low as ~60 CFUs/mL in water). Moreover, this platform exhibits potential as a versatile detection system for a diverse range of molecular and cellular analytes, thereby demonstrating its capacity for broader applications. Its profound advantages include a rapid analysis, cost effectiveness, and heightened sensitivity.

#### 2.5.6. *Yersinia pseudotuberculosis*

*Yersinia pseudotuberculosis*, a food and waterborne bacterium, is primarily transmitted among humans through the fecal–oral route, resulting in the development of yersiniosis [99,100,101]. Reactive arthritis, Reiter’s syndrome, erythema nodosum, glomerulonephritis, myocarditis, and sepsis are among the notable complications that can arise from *Yersinia pseudotuberculosis* infection [102]. Yang and colleagues have developed a novel colorimetric system for the detection of *Yersinia pseudotuberculosis*, utilizing phage particles and Au@Pt nanozymes. Initially, they isolated a highly specific phage (vB_YepM_ZN18) that targets *Y. pseudotuberculosis* from hospital sludge. Subsequently, they synthesized and immobilized Au@Pt nanozymes with peroxidase-like activity onto the surface of the isolated phage. Concurrently, another set of phages was modified with magnetic beads (MB). Additionally, a mixture of phages@MB and AuPt@phages was introduced into the bacterial samples for co-incubation. In the presence of the targeted bacteria, the phages@MB and AuPt@phages selectively adhered to the bacterial cells through specific interactions with the phage. After magnetic separation, the AuPt@phages bound to bacterial cells catalyzed the reaction between TMB and H_2_O_2_, producing a chromogenic solution. The lack of precise recognition in the presence of non-target bacteria facilitates easy removal of AuPt@phages along with the supernatant. His phage-based detection approach demonstrates outstanding performance characteristics and holds immense potential for the rapid identification of pathogenic microorganisms in diverse settings, such as drinking water, food production, and hospital samples [103]. This innovative approach, which integrates phages and Au@Pt nanozymes, provides a reliable and highly efficient colorimetric system for the precise detection of Yersinia pseudotuberculosis. It demonstrates significant potential for diverse applications in various fields, including monitoring drinking water safety, ensuring food hygiene, and accurately diagnosing infections in hospital environments.

### 2.6. Application of Nanozymes in Virus Detection

Viruses possess remarkable resilience and exhibit high transmissibility. Furthermore, their capacity for rapid mutation and genetic recombination significantly increases the likelihood of a pandemic, particularly in the context of an intricately interconnected globalized society [104]. A plethora of viruses, including influenza viruses, human immunodeficiency virus (HIV), Ebola virus (EBOV), Zika virus (ZIKV), and coronaviruses, have had significant impacts on public health throughout history. These effects range from the historical smallpox epidemic in the Aztec Empire in 1520 to the ongoing global COVID-19 pandemic [105]. In view of the ongoing COVID-19 pandemic, the pressing demand for rapid and highly sensitive detection methodologies targeting pathogenic agents has been further emphasized [106].

#### 2.6.1. HEV

Hepatitis E virus (HEV) infection has emerged as a significant public health concern, characterized by an increasing prevalence of Hepatitis E outbreaks in both developing and developed nations. The transmission of HEV primarily occurs through waterborne and zoonotic pathways, further emphasizing the seriousness of the situation [107]. Currently, significant progress has been achieved in the field of HEV detection platforms. These advancements mainly involve the application of RNA-targeting reverse transcription polymerase chain reaction (RT-PCR) techniques and the identification of specific antibodies (IgG, IgM, and IgA) generated as a result of HEV infection [108]. Due to the limited availability of diagnostic assays for the HEV, there is a pressing need for a reliable immunoassay targeting HEV antigens to effectively combat emerging outbreaks through early detection of the causative agent. In response to this demand, Khrois et al. have developed an innovative immunoassay platform utilizing nanozymes. The nanozymes are composed of anti-HEV IgG-antibody-conjugated gold nanoparticles (Ab-AuNPs) as the core, with an outer shell formed with in situ silver deposition on the surface of Ab-AuNPs. The virus is entrapped within these nanocomposites, while the silver shell can be decomposed into silver ions (Ag^+^) upon the introduction of tetramethyl benzidine (TMBZ) and H_2_O_2_. This decomposition process indirectly quantifies the concentration of the target virus. By incorporating the enhanced effect of the Ag shell on the AuNP-based nanozyme, the immobilization of advanced deposition has been verified to validate the proposed immunoassay’s signal amplification mechanism. This decomposition process indirectly measures the concentration of the target virus. The incorporation of an Ag shell in conjunction with an AuNP-based nanozyme enhances its effectiveness, and advanced deposition immobilization has been confirmed to validate the proposed immunoassay’s signal amplification mechanism. The implemented assay for Hepatitis E virus-like particles (HEV-LPs) demonstrated remarkable sensitivity, capable of detecting concentrations as low as 10 pg/mL within a linear range of 10 pg/mL to 10 ng/mL [109]. The proposed methodology possesses the potential to operate as a highly efficient surveillance system for virus detection. In conclusion, the advancement of this sophisticated immunoassay utilizing the nanozyme platform demonstrates significant promise in facilitating the identification and monitoring of HEV outbreaks, thereby making substantial contributions to enhancing public health outcomes.

#### 2.6.2. Nov-LP

Norovirus (NoV) is a genetically diverse group of small, icosahedral, non-enveloped viruses with a positive-sense, single-stranded RNA (ssRNA) genome [110]. NoV demonstrates a significant level of contagion primarily attributed to its exceptionally low infective dose, with the estimated median infective dose (ID_50_) ranging between 18 and 1015 genome copies [111]. Currently, the most reliable and sensitive approach for detecting and quantifying NoV is the reverse-transcription–quantitative-polymerase-chain-reaction (RT-qPCR) [112]. Detecting NoV in food poses significant challenges, requiring a virus concentration specific to the matrix and removal of inhibitory compounds for detection assays. As a result, the utilization of RT-qPCR faces obstacles in rapid in-field or point-of-care diagnostic applications. To address this issue, Weerathunge et al. propose a novel colorimetric nanozyme aptasensor strategy capable of enabling rapid (within 10 min) and highly sensitive detection of the infectious murine norovirus (MNV), with a calculated LOD of 3 viruses per assay (equivalent to 30 viruses/mL) and an experimentally demonstrated LOD of 20 viruses per assay (equivalent to 200 viruses/mL) [112]. The implementation of this novel assay format has significant implications, enabling rapid and highly sensitive norovirus detection within minutes. Additionally, it is user-friendly and eliminates the need for specialized laboratory facilities. The development of this colorimetric nanozyme aptasensor strategy represents a significant advancement in norovirus detection.

#### 2.6.3. RV

**Figure 6:**
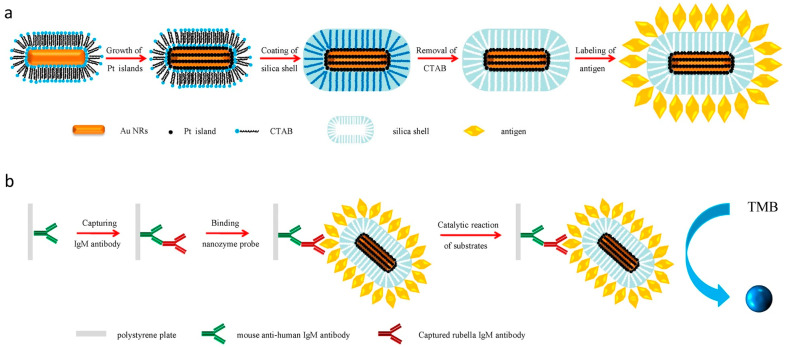
Figure (**a**) depicts the synthetic procedure employed for creating the antigen-labeled Au@Pt@SiO_2_ nanozyme. The schematic illustrates the sequential steps involved in synthesizing this nanozyme, highlighting the incorporation of antigen labels onto the surface of Au@Pt@SiO_2_ nanoparticles. Figure (**b**) elucidates the detailed process of conducting an immunoassay using the antigen-labeled Au@Pt@SiO_2_ nanozyme-based enzyme-linked immunosorbent assay (ELISA) system. The diagram clarifies key stages of this immunoassay, including target antigen immobilization, specific antibody recognition and binding, signal generation through nanozyme catalytic activity, and the subsequent quantification or analysis of generated signals (reproduced from Li et al. (2019) [118] under the Creative Commons CC BY license).

The rubella virus (RV), a human pathogen, is responsible for the occurrence of German measles, an airborne childhood disease renowned for its high contagiousness and global prevalence. When rubella infection occurs during pregnancy, it leads to the development of congenital rubella syndrome, which encompasses the well-established triad of cataracts, cardiac abnormalities, and sensorineural deafness [113,114]. Due to the importance of detecting the rubella virus, it is essential to use highly sensitive and effective detection methods. Among conventional approaches, using serological testing for rubella immunoglobulin (Ig) M is a standardized method for confirming acute infections [115,116]. Peroxidases, such as HRP, are widely employed in enzyme-linked immunosorbent assays (ELISAs) for the detection and quantification of antigens, antibodies, viruses, or cells. However, due to its inherent instability, HRP may result in a significant number of false-negative outcomes [117]. Taking inspiration from mesoporous silica-coated nanocrystals, which preserve the functional properties of their core and offer favorable surface functionalization characteristics, Li’s research group has developed a novel nanozyme denoted as Au-core@Pt-shell@mesoporous silica (Au@Pt@SiO_2_) for immunoassays. The synthesized Au@Pt@SiO_2_ nanozyme demonstrates catalytic capabilities in chromogenic reactions within immunoassays, thereby presenting a potential substitute for natural enzymes in conventional ELISAs. Additionally, a novel conjugate utilizing the antigen-labeled Au@Pt@SiO_2_ nanozyme has been designed as a nanoprobe for virus serodiagnoses. The antigen-labeled Au@Pt@SiO_2_ nanozyme exhibits highly sensitive peroxidase-like activity and remarkable catalytic stability and robustness, making it suitable for use in biochemical assays and clinical diagnoses (Figure 6) [118]. The research conducted by Li and colleagues, which focuses on the development of the Au-core@Pt-shell@mesoporous silica nanozyme and its utilization as a nanoprobe for virus serodiagnoses, represents a significant advancement in the field of rubella virus detection. The enhanced stability and catalytic properties demonstrated by this nanozyme offer significant potential for enhancing biochemical assays and clinical diagnoses, thereby promoting advancements in the accuracy and reliability of rubella virus detection and diagnoses.

#### 2.6.4. SARS-CoV-2

Since its emergence in late 2019, Severe Acute Respiratory Syndrome Coronavirus 2 (SARS-CoV-2) has spread globally, resulting in the development of a worldwide pandemic known as COVID-19 [119]. SARS-CoV-2, classified as an enveloped positive-sense single-stranded RNA virus, exhibits a high degree of contagiousness primarily through two modes of transmission: direct person-to-person contact and respiratory droplets [120]. In the context of a global epidemic outbreak, the primary area of concern lies in achieving a precise and expeditious diagnosis that encompasses characteristics such as simplicity and cost effectiveness [91]. Bradbury’s research team has developed an innovative paper-based device that integrates multiple components, including a lateral flow assay (LFA) test strip, dehydrated signal enhancement reagents (nanozymes and associated chemicals), and a sealed chamber containing a liquid enhancement buffer. These components are enclosed within a 3D-printed casing. The implemented device has demonstrated its efficacy by enabling the detection of N-protein in undiluted serum within a timeframe of 40 min, even at concentrations as low as 0.1 ng/mL. The device’s all-in-one design simplifies the process by requiring a single step to activate signal enhancement after the initial LFA detection [121].

To meet the requirements of POCT, Liu’s research team has developed a smartphone-based nanozyme-linked immunosorbent assay (SP-NLISA) for detecting SARS-CoV-2 NP antigens. Furthermore, the assay demonstrates exceptional sensitivity, enabling precise detection of NP antigens even at concentrations as low as 10 pg/mL. The simplicity, sensitivity, speediness, and cost effectiveness of this assay make it an excellent choice for detecting NP antigens in individuals suspected of SARS-CoV-2 infection [122].

Furthermore, Oh’s research team has developed a novel colorimetric assay called MagLISA, which is based on magnetic nanoparticles and nanozymes linked to immunosorbent assays. The aim of this technology is to achieve highly sensitive and precise detection of the influenza virus. This assay effectively combines newly synthesized superparamagnetic MagNBs and AuNZs (gold-nanoparticle-based nanozymes) to enhance peroxidase activity. The MagLISA integrates three essential components: MagNBs for biomarker enrichment, AuNZs as a peroxidase-like artificial catalyst, and an anti-hemagglutinin (HA) monoclonal antibody (mAb) for specific recognition facilitation. This innovative probe facilitates the identification, separation, and visualization of the influenza virus through a one-step colorimetric analysis. To evaluate the catalytic properties of the probe, molecular dynamics (MD) simulation is employed, considering their surface characteristics. The MagLISA demonstrates a significantly enhanced limit of detection (LOD) of up to 10–14 g·mL^−1^, particularly when combined with conventional commercial microreader equipment [123].

**Figure 7:**
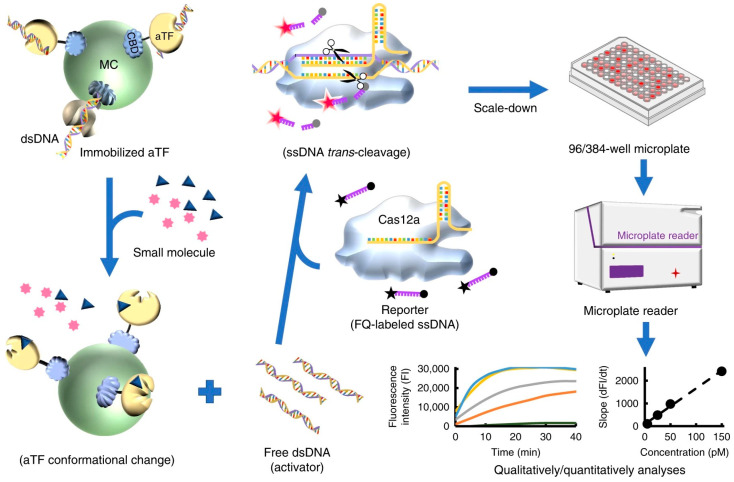
The schematic diagram depicts the CaT-SMelor system, encompassing multiple components and their interactions. These include MC (microcrystalline cellulose), aTF (allosteric transcription factor), CBD (cellulose-binding domain), dsDNA (double-stranded DNA), and FQ-labeled ssDNA (fluorophore-quencher-labeled single-stranded DNA). The diagram elucidates the interrelationships and functionalities of these constituents within the CaT-SMelor system (reproduced from Liang et al., (2019) [130] under the Creative Commons CC BY license).

Recently, CRISPR-Cas-based detection systems have emerged as a valuable approach for detecting various biological entities, including nucleic acids [124,125,126], proteins [127,128,129], and small molecules (Figure 7) [130,131,132]. The combination of fluorescence detection and the CRISPR-Cas system has become widely adopted as the primary methodology in this field. Wu’s research team has innovatively incorporated a colorimetric approach into their study by harnessing the catalytic properties of MnO_2_ nanozymes within a CRISPR-Cas12a system. The synthesized MnO_2_ nanorods demonstrate exceptional oxidase-like activity, facilitating an efficient catalytic reaction between TMB and the nanozymes that leads to a visible color transition from pale yellow to blue. These MnO_2_ nanorods are utilized as nanozyme labels for magnetic beads (MBs) through attachment via single-stranded DNA (ssDNA). In the presence of SARS-CoV-2, the activated Cas12a enzyme cleaves the ssDNA linker, inducing a color change for colorimetric detection. Notably, the MnO_2_-nanozyme-mediated CRISPR-Cas system provides a simplified alternative to traditional horseradish peroxidase (HRP)-catalyzed TMB-H_2_O_2_ systems by eliminating the need for hydrogen peroxide (H_2_O_2_) and exhibiting high robustness [22]. These studies present compelling evidence for the potential of colorimetric assays to achieve sensitive and specific virus detection. The incorporation of diverse nanomaterials, such as MagNBs and MnO_2_ nanorods, as peroxidase-like catalysts highlights the versatility offered by colorimetric methods in the field of virus detection. These innovative approaches provide valuable tools for rapid and cost-effective virus screening, showing great potential in their application to diagnostics and surveillance efforts.

#### 2.6.5. ZIKV

ZIKV, a member of the flavivirus (FLAV) genus, exhibits similarities with other members of this group, including dengue, chikungunya, and yellow fever viruses. As a result, distinguishing clinical symptoms associated with ZIKV infection from those related to these viral infections poses a challenge. ZIKV was first detected in Brazil in 2015 and rapidly spread globally, affecting over 50 countries across multiple regions including Central and South America as well as Southeast Asia [133,134]. The primary concern regarding ZIKV is centered on the increased risk of microcephaly and other neurological abnormalities observed in infants born to mothers who contracted the virus during pregnancy [135,136]. Hsu’s research team has pioneered the development of a point-of-care (POC) test for detecting ZIKV. This innovative test consists of an immunosensor vial integrated with platinum/gold core–shell nanoparticles (Pt@Au NPs), which are artificial nanozymes, and utilizes an iPhone 7 Plus smartphone. Remarkably, this POC test demonstrates a high level of specificity in detecting ZIKV in whole blood, exhibiting minimal cross-reactivity with other flaviviruses (such as dengue virus, DENV) during screening for Zika infection. The test procedure is designed to be user-friendly, allowing individuals to independently perform the Zika infection test without requiring any assistance from medical personnel. The procedure entails the straightforward application of a blood sample onto the vial immunosensor, which contains antibodies-modified Pt@Au NPs (Ab-Pt@Au NPs), followed by gentle agitation, capturing an image using the smartphone, and awaiting the automated assessment report generated by the computational algorithms embedded in the smartphone. This POC test is noteworthy for its ability to analyze whole blood without the need for filtration steps. Its user-friendly operation, combined with the quantification capabilities provided by a smartphone, make it highly suitable for deployment at ports of entry, airports, and other relevant locations frequented by travelers returning from endemic regions. Furthermore, this test shows potential for its application in surveillance efforts by enabling geographical tagging of infected individuals [137]. The innovative test has exceptional specificity in detecting ZIKV and is user-friendly, seamlessly integrating with smartphone technology. It serves as a valuable tool in fighting the spread of the ZIKV virus, especially in regions with limited access to conventional laboratories.

### 2.7. Plasmodium

Malaria is a prevalent infectious disease that has a significant global impact. According to data provided by the reputable WHO, there were an estimated 241 million reported cases of malaria in 2020 across 85 countries designated as malaria-endemic [138]. Timely diagnoses and prompt initiation of therapeutic interventions are crucial for effective disease management and minimizing malaria-associated mortality. Additionally, the development of precise detection methodologies is of paramount importance to reduce unnecessary administration of antimalarial medications and prevent the emergence of potential drug-resistant strains [139]. In a study conducted by Nicklen et al., Pt-coated Au nanozyme probes (PtGNPs) efficiently captured the target analyte, with the platinum coating exhibiting peroxidase-like enzymatic activity under low pH conditions. Notably, after biomarker preconcentration and signal enhancement, the LOD for pLDH was determined to be 0.01 ng/mL, representing a significant 1000-fold improvement compared to conventional LFA methodology [27]. It is crucial to emphasize that malaria remains a pressing global health issue, highlighting the urgency of expedited diagnoses and treatment. The LFA, due to its practicality, represents a feasible solution for effectively addressing challenges related to accessibility and time-to-result. The integration of platinum-coated gold nanozyme probes into LFAs has resulted in significant advancements in terms of enhancing sensitivity. This innovative approach not only enhances the accuracy of malaria detection but also shows promise for expanding the range of diagnostic applications toward identifying various biomarkers.

## 3. Nanozyme-Mediated Antibacterial Mechanisms

With the rapid development of nanozymes in the field of antibacterial mechanisms, this paper aims to provide a comprehensive overview and analysis of their physical and chemical properties, as well as their associated antibacterial mechanisms. The elucidation and synthesis of the antibacterial mechanisms facilitated by nanozymes have significant implications for the application and management of nanozymes in the fields of infectious disease control, combating biological fouling, and the development of related nanozyme-based biomedical technologies. Given the wide range of nanozymes available, their complex physical and chemical properties, as well as the presence of various interfering factors, it is crucial to systematically organize and summarize the universal antibacterial mechanisms. Drawing on recent research findings, we provide a comprehensive review of four key antibacterial mechanisms exerted by nanozymes: regulation of ROS, HOBr/Cl regulation, extracellular DNA clearance, and lysozyme and light-triggered carvacrol release.

### 3.1. ROS Regulation

**Figure 8:**
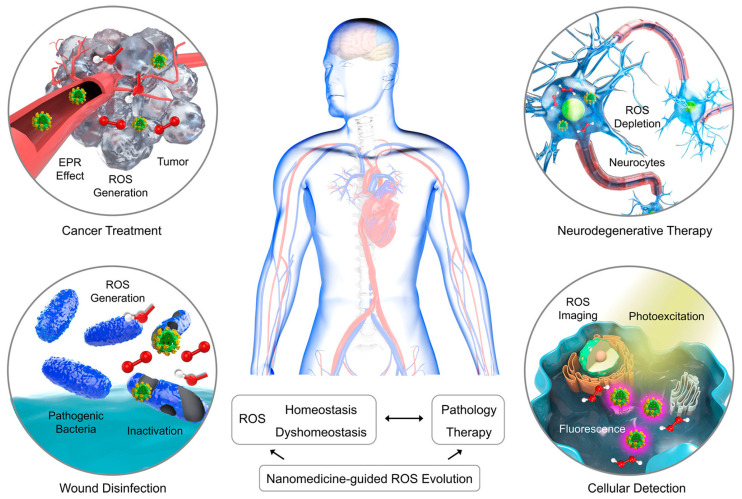
ROS-based nanomedicine has emerged as a promising strategy for diverse applications in the field of medical science. The unique material chemistry associated with nanomedicines confers distinctive capabilities in generating or depleting ROS, thereby facilitating the treatment of various pathological dysfunctions, including but not limited to cancer, neurodegenerative diseases, and bacterial infections. Concurrently, analytical technologies have been developed to evaluate the effectiveness of these therapeutic platforms in regulating ROS levels, ensuring accurate assessment of their ROS-modulating performance (reproduced from Yang et al., 2019) [152].

ROS is a comprehensive designation employed to describe a group of exceptionally reactive radicals that originate from unpaired electrons associated with oxygen, namely the hydroxyl radical (•OH) and superoxide (•O^2−^) [140]. In vivo, the main source of ROS is located at the substrate end of the mitochondrial inner membrane, where the electron transport chain complex facilitates the transfer of electrons from mitochondria to O_2_. As a result, a portion of O_2_ undergoes reduction, leading to the generation of either superoxide (O^2−^) or H_2_O_2_ [141]. ROS are widely recognized as deleterious metabolites capable of causing damage to cellular proteins, lipids, and nucleic acids. Simultaneously, ROS also serves as an effective phagocytic armamentarium, enabling the host to counteract invasive pathogens [142]. Despite their benefits, conventional antioxidants face challenges such as reduced stability, increased toxicity, and limited bioavailability [143] (Figure 8). In recent years, the progress of nanoscience and technology has introduced innovative antioxidant methodologies based on nanozymes, which have gained significant attention and implementation in combating ROS and their associated antibacterial effects (Table 1). The CeO_2_ nanozymes [144,145] have been demonstrated to operate through a detection mechanism that relies on the crystal structure of CeO_2_, which is characteristic of their catalytic activity. In the presence of bacterial suspensions, oxygen vacancies are created, resulting in the release of oxygen atoms accompanied by two valence electrons. These electrons can subsequently be captured by Ce^4+^ ions and suspended oxygen molecules, leading to the generation of Ce^3+^ ions and •O^2−^. The enhanced Ce^3+^/Ce^4+^ ratio promotes the manifestation of SOD-like activity through the following mechanism: •O^2−^ + Ce^3+^ + 2H^+^ →H_2_O_2_ + Ce^4+^ [146]. Furthermore, diverse strategies have been investigated to effectively enhance the production of ROS mediated by nanozymes, thereby augmenting their antibacterial efficacy. These approaches include promoting plasmon–exciton coupling [147], utilizing spherical mesoporous SAzyme materials [23], leveraging NIR-enhanced peroxidase activity [148], and incorporating enriched multi-porphyrin structures [30]. These methodologies collectively aim to enhance the antibacterial effect by boosting the generation of ROS through nanozyme-based interventions. Through manipulation of the initial substrate concentration (e.g., TMB or H_2_O_2_), a comprehensive set of enzyme kinetic parameters, including the Michaelis–Menten constant (K_m_) and maximum initial velocity (V_max_), can be determined. These parameters serve as critical indicators for evaluating the substrate binding affinity and catalytic efficiency of nanozymes [144,147,149,150,151]. Moreover, a diverse array of enzymes with enzyme-like activities, including REDOX nanozymes containing SOD, CAT, POD, laccase, hydrolytic nanozymes, cleavage nanozymes, and other component cascade catalysts, can be covalently linked to expand the spectrum of antibacterial properties and broaden the application domain. These integrated systems hold significant potential for combating biofilm formation and addressing inflammation associated with bacterial infections. In summary, ROS demonstrate exceptional reactivity, capable of causing damage to cellular components while simultaneously serving as an innate defense mechanism against pathogens. The conventional use of antioxidants encounters inherent limitations, thereby stimulating the pursuit of innovative antioxidant strategies founded upon nanozymes. Notably, CeO_2_ nanozymes, characterized by their distinctive crystal structure, have demonstrated considerable potential in bolstering ROS-mediated antibacterial undertakings. Moreover, alternative approaches involving plasmon–exciton coupling, mesoporous SAzyme materials, and NIR-enhanced peroxidase have been successfully explored, further enhancing the antioxidant capabilities of nanozymes.

### 3.2. HOBr/Cl Generation

**Figure 9:**
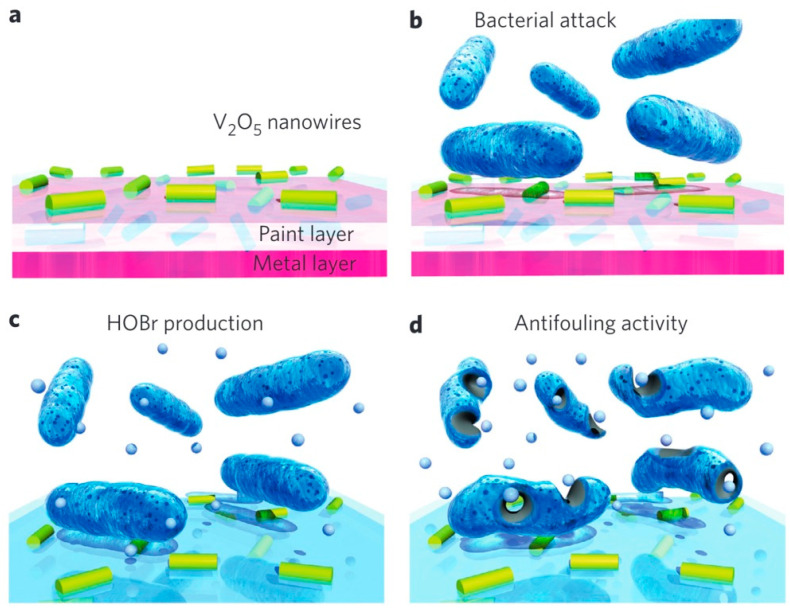
Antimicrobial activity of V_2_O_5_ nanowires. (**a**) When incorporated into a matrix (such as paint) and subsequently applied onto a metallic surface, the V_2_O_5_ nanowires exhibit bactericidal properties. The nanowires, depicted as yellow-green rods, are embedded within the matrix. (**b**) Upon encountering V_2_O_5_ nanowires, bacteria exhibit increased susceptibility and are more easily neutralized. (**c**) The V_2_O_5_/paint nanocomposite exhibits inherent biomimetic catalytic behavior similar to that of vanadium haloperoxidases (V-HPOs). In the presence of substrates such as Br_2_ and H_2_O_2_, small amounts of hypobromous acid (HOBr, represented as small light-blue spheres) are continuously generated. (**d**) The HOBr released as a result of the catalytic reaction disrupts the quorum-sensing mechanism employed by bacteria, thereby hindering bacterial adhesion and impeding the formation of biofilms (reproduced from Natalio et al., 2012) [158].

Biofouling, a significant environmental phenomenon, is commonly encountered in marine ecosystems, exerting adverse impacts on diverse forms of marine life [156]. Marine organisms constantly face the persistent risk of epibiont overgrowth, compelling them to develop intricate chemical or physical antifouling mechanisms [157]. Marine algae have developed sophisticated chemical mechanisms to combat microbial fouling by interfering with quorum sensing, a complex process in which bacteria assess cell density and regulate various phenomena, including biofilm formation (Figure 9) [158]. In modern times, the significance of seaweed and certain lichens has been elucidated in their pivotal role in combating biofouling. They achieve this by catalyzing the production of biocidal hypohalous acid HOX (with X denoting I^−^, Br^−^, or Cl^−^) through the oxidation of halide ions with H_2_O_2_ [159]. Halogenic acids possess the ability to effectively eliminate bacteria and biofilms, making them a crucial component in biological antifouling strategies. Currently, various nanozymes based on vanadium and cerium have demonstrated exceptional halogenated-peroxidase-like activity when combined with H_2_O_2_ [156,158,160,161] (Table 2). The identification of vanadium- and cerium-based nanozymes exhibiting elevated halogenated-peroxidase-like activity represents a promising approach towards the development of effective antifouling solutions, capable of disrupting biofilm formation and eliminating bacterial populations in diverse environments.

### 3.3. Extracellular DNA Clearance

**Figure 10:**
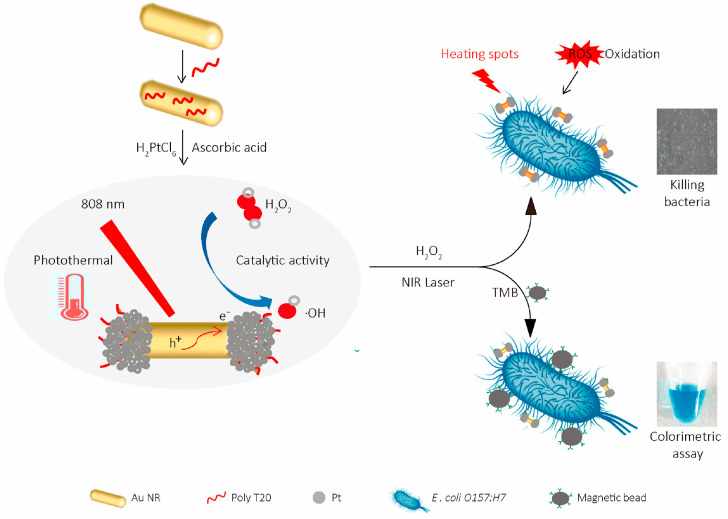
The provided information describes a schematic illustration of DNA-mediated Au-Pt nanoparticles that exhibit both photothermal characteristics and enhanced enzyme-like catalytic activity. These nanoparticles are further utilized for the eradication of *E. coli O157:H7* as well as for the colorimetric detection of the same bacterium (reproduced from Lu et al., 2021) [167].

Bacterial biofilms are complex multicellular communities enclosed within an extracellular polymeric substance (EPS), exhibiting robust structural integrity and serving as a mechanically resilient protective barrier. The intricate physical and biological characteristics inherent in biofilms contribute to their heightened resistance against conventional therapeutic interventions, thereby posing challenges in effectively managing infections associated with biofilms [162]. Upon exposure to traditional antibiotics, bacteria have a tendency to develop resistance, which not only perpetuates existing resistance but also exacerbates the overall challenge associated with effectively combating bacterial infections [8]. Simultaneously, the bacterial membrane plays a crucial role in the innate defense mechanisms of bacteria. However, antibiotics face limitations in penetrating the biofilm barrier, which restricts their antibacterial efficacy [163,164]. Currently, the utilization of endogenous deoxyribonuclease (DNase) has emerged as a promising strategy for mitigating biofilm formation by targeting the extracellular matrix (ECM) for removal [165]. Despite its potential as an antibacterial agent against biofilms, the implementation of natural DNase is hindered by certain drawbacks such as elevated costs, fluctuating catalytic activity, and irreversibility. To address this challenge, DNase nanozymes have emerged as a promising approach to effectively target and remove DNA in combating biofilms (Table 3). The nanozymes effectively overcome the limitations associated with expensive and unstable natural enzymes, demonstrating their ability to penetrate the biofilm matrix, strongly inhibit biofilm formation, and thus exert a rapid bactericidal effect [166] (Figure 10).

Furthermore, from a broader perspective, DNase exhibits remarkable capabilities in rapidly disrupting the structural integrity of the ECM and significantly enhancing its sterilization efficacy when compared to conventional antibiotics. This effective approach is particularly valuable in combating drug-resistant bacterial strains and has extensive potential for various applications. Present in both natural and nanozyme forms, DNase exhibits a broad spectrum of sterilization activity, surpassing that of traditional antibiotics. As a result, its application demonstrates significant potential and extensive utility in combating infections associated with biofilm formations.

### 3.4. Lysozyme and Light-Triggered Carvacrol Release

Nanozymes, renowned for their exceptional catalytic efficiency, have demonstrated the ability to synergize with other bactericidal agents, thereby exhibiting a more potent multifaceted bactericidal effect. Nong’s research team has reported the development of a metal–organic framework-based nanozyme hybrid that showcases coordinated actions of lysozyme and light-triggered carvacrol release in eradicating bacterial infections. This innovative nanozyme hybrid encompasses a range of functionalities, including magnetic aggregation, photothermal conversion, electrostatic attractions that facilitate bacterial capture, enzymatic hydrolysis, and light-controlled carvacrol release [171]. The synergistic aggregation of nanozymes and complementary bactericidal substances represents a highly promising approach for the development of a broad-spectrum bactericide, exhibiting remarkable potential in the domains of biomedicine, food safety, and environmental protection. This strategy holds significant promise for achieving comprehensive bactericidal properties. The applicability of nanozymes extends to various fields, such as biomedicine, food safety, and environmental protection. The integration of nanozymes with distinct bactericidal mechanisms provides new opportunities for enhancing bacterial eradication effectiveness and addressing the challenges associated with antibiotic resistance.

## 4. Applications of Nanozymes in Antibacterial Mechanisms

In recent years, the exploration of novel class substitutes possessing mimetic functions has become a prominent research area, inspired by the remarkable attributes exhibited by natural enzymes. In this regard, significant attention has been garnered by various nascent nanomaterials collectively referred to as nanozymes due to their unexpected and compelling enzyme mimetic activities [37]. The robust antibacterial efficacy demonstrated by nanozymes is particularly noteworthy, as it has sparked the exploration of diverse innovative antibacterial strategies [172]. Within this section, our objective is to present a comprehensive compilation of exemplary nanozymes and their associated antibacterial strategies, categorized based on their elemental composition.

### 4.1. Metal-Based Nanozymes

#### 4.1.1. Iron-Based Nanozymes

**Figure 11:**
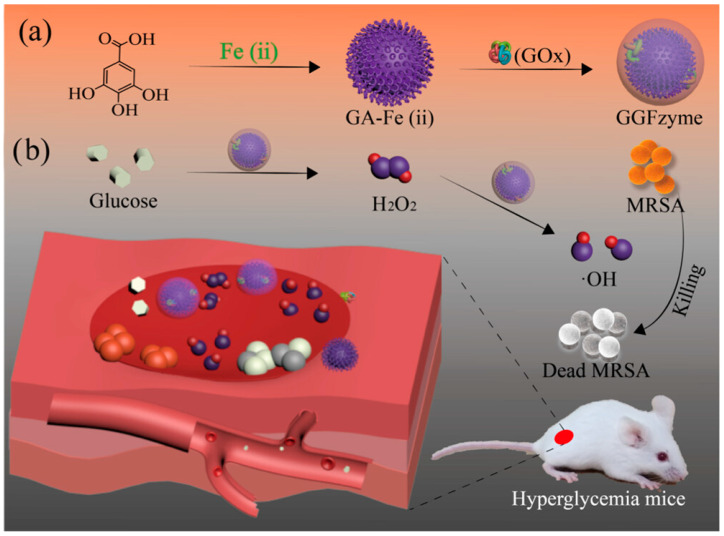
Figure (**a**) illustrates the synthesis process employed for GGFzyme, showcasing the sequential steps involved in its creation using a specified methodology. Figure (**b**) demonstrates the application of GGFzyme in inhibiting MRSA infection in hyperglycemic mice with wounds. The diagram depicts the specific intervention and its impact on reducing MRSA infection near the wounds in these mice exhibiting hyperglycemia (reproduced from Shi et al., 2022) [182].

The utilization of iron-based nanomaterials has attracted significant attention in the field of biomedical applications, primarily due to their distinct paramagnetic or superparamagnetic magnetization characteristics. A Fe_3_O_4_@MoS_2_-Ag nanozyme with an abundant defect rough surface was prepared using a simple hydrothermal method and in situ photo deposition method, demonstrating a good bacteriostatic effect (~69.4%) against *Escherichia coli*. Additionally, the magnetic recovery of Fe_3_O_4_ provides power for efficient disinfection of the nanozyme [173]. Drums et al. developed a cost-effective and versatile approach by utilizing paramagnetic iron oxide particles (SPION) conjugated with fructose metabolites, which effectively enhanced the uptake and therapeutic efficacy of SPION against both Gram-positive MRSA and Gram-negative biofilms (e.g., *E. coli* and *Pseudomonas aeruginosa*) [174]. These materials have demonstrated immense potential in various biomedical domains, including bio-separation, targeted drug delivery, magnetic resonance imaging, biosensors, and hyperthermia therapy [173,175,176]. In 2007, Gao and Yan presented pioneering evidence that magnetite (Fe_3_O_4_) nanoparticles possess inherent peroxidase-like enzymatic activity due to the substantial presence of Fe^2+^ and Fe^3+^ ions on their surface [53]. This pioneering research has served as a catalyst for the exploration and subsequent implementation of iron-based nanozymes in diverse biological domains, encompassing bioassays, tumor therapy, and antibacterial interventions [177,178,179]. The research conducted by Sun’s group has involved developing an enzymatic antibacterial approach centered around thermogenic nanozymes, which aims to achieve synergistic bacterial inhibition by enhancing the activity of nanozymes while simultaneously reducing the activity of natural enzymes in bacteria [29]. Specifically, yolk-shell structured Fe_2_C@Fe_3_O_4_ NPs with a uniform size of approximately 20 nm were synthesized as the thermogenic nanozymes, exhibiting exceptional magnetothermal efficiency and escalated peroxidase-like activities facilitated by thermal enhancements [29]. Furthermore, in order to achieve an elevated level of nanozyme activity, a Fe,N co-doped ultrathin hollow carbon framework (Fe,N-UHCF) was synthesized and characterized by the substantial presence of Fe-Nx bonding as well as its distinct morphology. The Fe,N-UHCF framework exhibits exceptional peroxidase-like activity, surpassing that of nearly all previously reported nanozymes [180]. Ali and his colleagues conducted a study on the fabrication of Fe-doped MoS_2_ (Fe@MoS_2_) nanomaterials, which exhibited an enhanced peroxidase-like activity through a co-catalytic mechanism. The remarkable improvement can be attributed to the synergistic effect resulting from the concurrent co-catalytic activities of Fe and MoS_2_, as well as the inherent characteristics of MoS_2_ layers [178]. Furthermore, Liao and colleagues have achieved a significant breakthrough by engineering FePO_4_-hydrogel (FePO_4_-HG) as an exceptionally effective antibacterial therapeutic approach. The FePO_4_ nanoparticles (NPs) were synthesized using the hydrothermal growth method and subsequently modified with L-cysteine to produce FePO_4_-Cys. These were then covalently linked to the hydrogel (HG) through an amidation reaction, resulting in the fabrication of FePO_4_-HG. This novel construct exhibits notable trienzyme-like functionality, eradicating bacteria through peroxidase-like catalytic capabilities while safeguarding normal cells from exogenous H_2_O_2_-induced damage via synergistic antioxidant effects stemming from the superoxide dismutase- and catalase-like activities of FePO_4_-HG [181]. A multifunctional GGFzyme was successfully synthesized using a one-pot method, effectively and promptly combining glucose oxidase (GOx), gallic acid (GA), and ferrous ions (Fe^2+^). This innovative GGFzyme utilizes endogenous glucose molecules in the local environment to generate a controlled cascade of ROS, acting as a cytotoxic agent against MRSA commonly found in wound surroundings with high mortality rates. Notably, this approach eliminates the need for exogenous H_2_O_2_ administration. By efficiently reducing glucose concentration, GGFzyme promotes simultaneous elimination of pathogenic bacteria and wound healing, offering a promising therapeutic strategy for hyperglycemic wound treatment (Figure 11) [182]. In summary, these investigations highlight the vast potential of iron-based nanozymes in various biomedical fields, offering innovative solutions for bioassays, tumor therapy, antibacterial interventions, and other emerging biomedical challenges.

#### 4.1.2. Cu-Based Nanozymes

**Figure 12:**
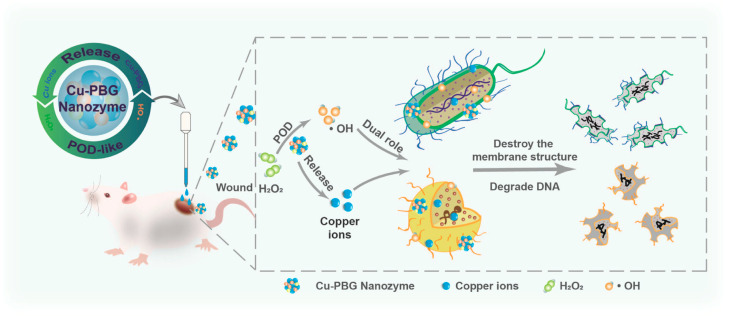
Schematic illustration of Cu-PBG-mediated antibacterial therapy (reproduced from Liu et al., 2021) [186].

Copper (Cu), a crucial microelement in living organisms, plays a significant role in the redox processes of native enzymes, as demonstrated by Cu-Zn superoxide dismutase and tyrosinase [183,184]. In parallel, although Cu ions have demonstrated commendable antibacterial efficacy, their significant toxicity limits direct utilization within living organisms [37]. Currently, investigations have confirmed the more favorable applicability of Cu-based nanomaterials as nanozymes due to their broader catalytic activity pH range compared to their Fe-based counterparts [37]. Drawing inspiration from natural enzymes’ active site structure, Liang and colleagues used Cu^2+^ to functionalize melanin nanoparticles (NMPs) in cuttlefish-ink-derived ink. This approach produced a highly stable and active SOD-like nanozyme (Cu-NMPs) with strong free radical scavenging capabilities. Subsequently, the simulated enzyme was incorporated into carrageenan films for food packaging purposes. Results showed that at a concentration of 10 μg/mL, Cu-NMPs generated over 80% •O^−^_2_ and exceeded natural SOD enzyme activity by reaching 60 U/mL [185]. The research group, led by He, developed a versatile nanozyme called polydopamine (PDA)-modified copper oxide (Cu_x_O-PDA). Cu_x_O-PDA demonstrated peroxidase-like behavior, which was further enhanced under near-infrared (NIR) irradiation. Under neutral or alkaline conditions, Cu_x_O-PDA had a negative surface charge and showed minimal peroxidase activity. However, under acidic conditions, the surface charge of Cu_x_O-PDA can become positive, allowing for specific targeting of negatively charged bacteria. Interestingly, well-dispersed Cu_x_O-PDA quickly aggregates upon NIR irradiation, trapping bacteria and nanozymes in close proximity. The study revealed that a decrease in the distance between the nanozyme and bacteria led to an increase in antibacterial efficacy. The conducted experiments demonstrated that the Cu_x_O-PDA nanozyme induced DNA degradation, lipid peroxidation, and eradication of biofilm formations [170]. Liu et al. developed and synthesized biodegradable Cu-doped phosphate glass (Cu-PBG) nanozymes, which exhibit potent antibacterial activity against both Gram-positive and Gram-negative bacteria. The antimicrobial mechanism of Cu-PBG involves the generation of ROS and copper release. In wounds, it functions akin to peroxidase by inducing lethal oxidative stress on bacteria by catalyzing the decomposition of H_2_O_2_ into •OH. Additionally, Cu-PBG possesses inherent degradability attributed to its phosphate glass properties (Figure 12) [186]. These studies emphasize the potential of Cu-based nanomaterials as nanozymes, providing a promising alternative for various applications such as food preservation and antibacterial interventions. Their unique characteristics, including a wide pH range and enhanced catalytic efficacy under specific circumstances, make them invaluable tools in both biomedical and environmental fields.

#### 4.1.3. Noble Metal-Based Nanozymes

Due to their notable surface metal atom ratios, various noble metal-based nanozymes, including Au [167], silver [169], platinum [187], and palladium [176], exhibit substantial catalytic activity. As a consequence, these nanozymes have found widespread utilization in the realm of biomedical applications, specifically in biosensing ventures [188]. Previous studies have predominantly observed monometallic compositions at the branch ends of dendritic structures. It is well-established that the peroxidase-like activity exhibited by monometallic nanozymes consistently falls short compared to their multimetallic counterparts [189,190]. According to the Sabatier principle, it is hypothesized that maintaining an optimal level of catalyst–intermediate interaction is crucial for achieving a balanced and moderate interaction between the catalyst and intermediate species [191,192]. Alloying is a suitable strategy for finely tuning the interaction between the catalyst’s surface and intermediate species. Pd nanocrystals have been shown to exhibit facet-dependent oxidase and peroxidase-like activities, endowing them with exceptional antibacterial efficacy through the generation of reactive oxygen species (ROS) [193]. Sun’s research group successfully developed a nanoplatform, designated as Pd@Pt-T790, which was easily synthesized by conjugating enzyme-catalytic Pd@Pt nanoplates with the organic sonosensitizer meso-tetra(4-carboxyphenyl) porphine (T790). Notably, it was observed that the incorporation of T790 onto Pd@Pt resulted in a significant suppression of the catalase-like activity exhibited by Pd@Pt. Under ultrasonic irradiation, the nanozyme activity was effectively restored, facilitating the catalytic decomposition of endogenous H_2_O_2_ into O_2_. This “blocking and activating” phenomenon is crucial in mitigating potential toxicity and adverse effects of nanozymes on normal tissues, while also offering significant potential for achieving active, controllable, and disease-targeted nanozyme catalysis [188]. The development of ultrasonic (US)-switchable nanozyme systems represents a highly promising avenue for enhancing the active, controlled, and precise eradication of deeply entrenched bacterial infections through sonodynamic modalities. This innovative strategy demonstrates remarkable potential for future prospects in the realms of biomedical research and clinical therapeutics, laying the foundation for transformative applications.

Simultaneously, Yang et al. employed Cu,I-doped carbon dots (Cu, i-CDs) as a reducing agent in conjunction with nanozymes to fabricate an Au-based nanozyme composite material (AuNPs/Cu,I). The AuNPs/Cu,I nanozymes not only emulate the intrinsic activities of superoxide dismutase, peroxidase, and catalase under diverse conditions but also serve as surface-enhanced Raman spectroscopy (SERS) enhancers. The integration of Cu, I-CDs, and AuNPs enhances electron transferability, leading to augmented peroxidase-like activity and superoxide-like activity. The multi-enzyme-like functionality of AuNPs/Cu,I nanozymes can be precisely modulated by altering the composition of Cu^0^/Cu^+^ and Au [194]. In addition, Prasad’s team synthesized bimetallic Cu-M nanoparticles (M = Au, Ag, Pt, or Pd) using an electrical substitution (GR) reaction. The catalytic activity of the bimetallic system was compared with that of Cu nanozymes, and the Cu-Pt nanozymes showed the highest catalytic efficiency [195]. The combination of both materials takes advantage of the two properties of the redox processes of native enzymes of Cu and the significant catalytic activity of novel metal for applications of nanozymes in antibacterial mechanisms.

#### 4.1.4. Vanadium-Based Nanozymes

Apart from the previously mentioned nanozymes based on Fe, Cu, and noble metals, vanadium (V)- and cerium (Ce)-based nanomaterials, possessing intrinsic mimic enzyme catalytic properties, are commonly denoted as vanadium-based nanozymes [37]. The exceptional capacity of vanadium (V)-based nanomaterials to mimic multiple enzymatic activities has been well-documented, demonstrating their efficacy in combating bacterial infections. These nanomaterials exhibit remarkable potential for addressing such infections due to their superior multienzyme-mimicking properties [196]. Ma et al. successfully developed a highly efficient bienzyme system, which demonstrated potent synergistic antibacterial activity. Vanadium oxide nanodots (VO_x_NDs) were synthesized using a streamlined ethanol–thermal strategy with vanadium (III) trichloride (VCl_3_) as the precursor. The as-synthesized VO_x_NDs exhibited remarkable peroxidase- and oxidase-like activities, mimicking natural enzymes’ functions. This antibacterial system showed exceptional efficacy against both non-resistant bacteria, including Gram-positive *S. aureus* and *Gram-negative E. coli*, and drug-resistant strains such as ESBL-producing *E. coli*, *MRSA*, and kanamycin-resistant *E. coli*. Importantly, due to their nanoscale dimensions, the VO_x_NDs displayed favorable biosafety as confirmed with various assessments including MTT assays, examination of physiological indices, and H&E staining [196]. Hu’s research team successfully synthesized a targeted nanozyme, Co^II^TBPP(bpy), using meticulous supramolecular self-assembly techniques. This unique nanozyme possesses peroxidase-like properties and can generate •OH radicals without light, showing exceptional production of ROS under irradiation. This causes bacterial membrane disruption and the release of intracellular contents, resulting in synergistic antibacterial effects. Co^II^TBPP(bpy) also efficiently decomposes excess H_2_O_2_ into O_2_, thanks to its outstanding catalase-like activity. The catalytic process improves PDT efficacy, and reduces tissue hypoxia and excessive H_2_O_2_ levels. The synthesized nanozyme shows good biocompatibility and achieves over 95% antibacterial efficiency in vitro. The supramolecular self-assembly of these multi-porphyrin structures offers great potential for use in antibacterial treatments and wound healing [150]. Furthermore, vanadium- and cerium-based nanomaterials have emerged as promising candidates for mimicking haloperoxidase activity to address the challenge of low stability in natural enzymes. These nanomaterials possess the capability to mitigate biofouling and enable the development of materials with prolonged stability even in demanding environments [197]. Hu’s research group developed free-standing nanofibrous mats using electrospinning PVA with CeO_2−x_ NRs. After cross-linking, the resulting PVA mats incorporated CeO_2−x_ NRs and exhibited haloperoxidase-like activity in water environments. The CeO_2−x_ activity within the PVA fibers was quantitatively assessed with bromination of phenol red, confirming that the nanozyme remained active even after being processed in the polymer matrix. These mechanically robust hybrid mats exhibited catalytic oxidation of Br^−^ and H_2_O_2_ to HOBr, similar to natural haloperoxidases. Leveraging the haloperoxidase-like activity of ceria NRs in PVA mats, along with their easy production, these PVA/CeO_2−x_ hybrid fibrous networks show promise for robust anti-biofouling coatings on surfaces exposed to humid and marine environments [160]. The present study elucidates the inherent potential exhibited by nanomaterials derived from vanadium and cerium as nanozymes, thereby offering a wide range of applications including antibacterial therapies, wound healing, and the fabrication of durable materials tailored for demanding conditions. The unique catalytic properties and excellent compatibility with biological systems make these nanomaterials highly promising candidates for future biomedical advancements and environmental initiatives.

### 4.2. Carbon-Based Nanozymes

Recently, carbon-based nanomaterials have emerged as highly promising entities in the field of biomedicine due to their exceptional physicochemical properties, commendable biocompatibility, cost effectiveness, and versatile mimicry of multiple enzyme functionalities [37]. Numerous efforts have been dedicated to exploring the antibacterial potential of carbon-based nanomaterials. Fang et al. successfully synthesized and modified a variety of oxygenated nanodiamonds (O-NDs) with different oxidation degrees using a simple yet efficient mixed acid-assisted reflux method, giving them remarkable peroxidase mimicking capabilities. A thorough spectroscopy analysis and chemical structure characterizations conclusively revealed the presence of carbonyl, carboxyl, and nominal hydroxyl groups in these O-NDs. Consequently, these nanomaterials exhibited enhanced enzymatic activity across a wide pH range. Importantly, spectroscopic observations and structural investigations suggested a significant increase in enzymatic activity during prolonged experimental durations. Furthermore, the addition of trace amounts of H_2_O_2_ resulted in exceptional antibacterial efficacy demonstrated by O-NDs, effectively eliminating periodontal bacteria and disrupting biofilms under physiologically relevant conditions both in vitro and in vivo [198]. Carbon-based nanomaterials, particularly oxygenated nanodiamonds, have shown great potential in the field of biomedicine due to their exceptional properties and surface modifications that enhance enzymatic activity and potent antibacterial effects. These significant advancements offer promising avenues for exploring novel antibacterial strategies and therapeutic interventions in biomedicine.

### 4.3. Transition Metal-Based Nanozymes

Transition metal nanomaterials have attracted significant attention due to their exceptional photo-thermal and electronic properties, as well as their commendable biosafety profile. These characteristics make them highly valuable and extensively utilized in various domains including sensing, imaging, catalysis, and biomedicine [199,200]. Bai’s research team developed a new heterostructure called ZnO@GDY NR, which consists of zinc oxide nanorods layered with graphdiyne nanosheets. This innovative configuration acts as a piezocatalytic nanozyme, where the graphdiyne layer provides electrical conductivity, adsorption, and catalytic nanozyme activity, while the zinc oxide layer is responsible for piezo catalysis and catalytic nanozyme activity. The resulting piezocatalytic nanozyme shows impressive antibacterial efficacy, with nearly 100% effectiveness against drug-resistant pathogens such as methicillin-resistant *Staphylococcus aureus* and *Pseudomonas aeruginosa* in both in vitro and in vivo settings [153]. The present investigation not only demonstrates the exceptional antibacterial efficacy of the ZnO@GDY NR heterostructure as a nanozyme but also highlights its commendable biocompatibility characteristics. These valuable observations not only contribute essential knowledge but also serve as a pivotal reference for the conceptualization and advancement of forthcoming antibacterial nanozymes that prioritize enhanced biocompatibility, thereby fostering their potential in biomedical applications.

### 4.4. Single-Atom Nanozymes

**Figure 13:**
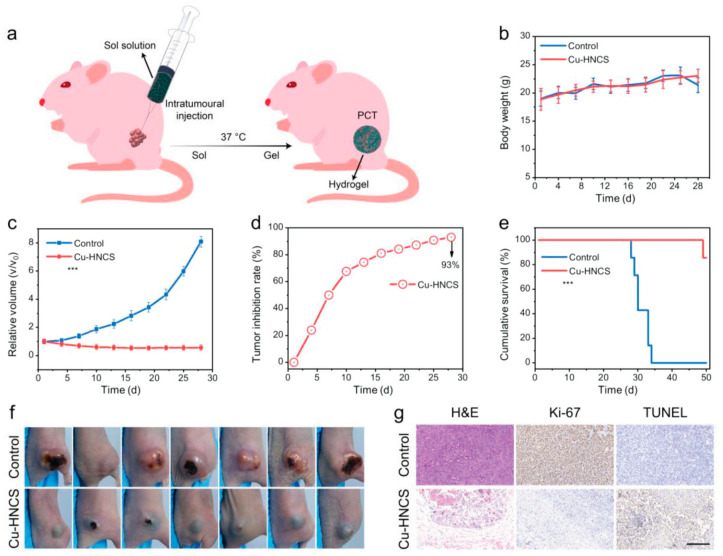
Parallel catalytic therapy was conducted in a subcutaneous tumor model. The study provided (**a**) schematics illustrating intratumoral administrations and the formation of an injectable hydrogel. (**b**) Body-weight curves, (**c**) tumor proliferation curves, (**d**) relative tumor inhibition rates, and (**e**) survival curves of BALB/c nude mice treated with blank hydrogels or Cu-HNCS hydrogels were presented. Data were statistically analyzed using Student’s *t*-test and reported as the mean ± SD (n = 7 per group). Statistical significance levels were denoted as *** *p* < 0.01. (**f**) Photographs depicting the state of 4T1 tumors after different treatments at day 28 were included. (**g**) H&E staining was performed to assess nuclear dissociation, Ki-67 immunofluorescence staining was used to evaluate cellular proliferation, and TUNEL staining was employed to detect necrosis of tumor cells in tumor sections (scale bars: 400 μm) (reproduced from Lu et al., 2020) [203].

Controlling metal species at the atomic level is a highly effective strategy for enhancing both the efficiency of metal utilization and the catalytic performance of nanomaterials [201]. In 2011, Qiao and his research group introduced the innovative concept of single-atom catalysts (SACs), which has since revolutionized the field of catalysis [202]. Following their creation, SACs have gained significant attention in the field of catalysis due to their advantages including efficient atom utilization, high catalytic activity, exceptional stability, and remarkable selectivity [34]. Recently, a group of SACs have emerged as single-atom nanozymes (SAzymes), exhibiting atomically dispersed active sites and coordination environments comparable to those observed in natural metalloenzymes. This novel development not only provides valuable insights into the catalytic mechanisms of nanozymes but also establishes a crucial connection between natural enzymes and nanozymes [37]. SACs exhibit strong catalytic ability and allow for efficient use of metals, which is particularly important in cancer and antibacterial treatments. This capability ensures effective therapeutic outcomes even at low metal concentrations (Figure 13) [203,204]. N-doped porous carbon (NPC) has gained widespread recognition as a specialized nanoplatform for constructing SACs, thanks to its commendable catalytic efficacy, outstanding biosafety profile, distinctive mesoporous structure, and substantial specific surface area. In this study, they present the synthesis of Cu single-atom sites/NPC (Cu SASs/NPC) via a PEAP strategy, which exhibits notable nanozyme properties for photothermal–catalytic antibacterial therapy. The Cu SASs/NPC composite demonstrates superior catalytic activity, glutathione-depleting capabilities, and photothermal effects compared to pure NPC without Cu doping. These Cu SASs/NPC specimens, being peroxidase-like nanozymes, efficiently catalyze the conversion of H_2_O_2_ into •OH, facilitating conspicuous antibacterial efficacy. Additionally, under laser irradiation, the photothermal effect of Cu SASs/NPC enhances the peroxidase-like catalytic function, driving increased production of ROS and consequently yielding enhanced in vitro antibacterial effects [205]. In addition, Wang’s research group successfully synthesized a Mo SA-N/C single-atom nanozyme with remarkable heterogeneous peroxalate (HPO)-mimicking activity and visible-light-responsive photothermal behavior, offering a promising approach to mitigate biofouling in seawater environments. As an HPO-like nanozyme, Mo SA-N/C effectively catalyzes the oxidation of bromide ions (Br^−^) to produce cytotoxic hypobromous acid (HOBr). Furthermore, the application of visible light irradiation significantly enhances the oxidation activity of Br^−^ on the surface of Mo SA-N/C through a pronounced photothermal effect. To better understand how Br- is oxidized, we conducted DFT calculations. Ultimately, Mo SA-N/C showed excellent antibacterial properties when exposed to both Br- and H_2_O_2_, as well as impressive performance in preventing biofouling. This accomplishment opens up possibilities for the advancement of biocompatible and photo-responsive synthetic enzymes, expanding our capacity to combat marine biofouling [206]. The application of Cu single-atom sites/N-doped porous carbon (Cu SASs/NPC) as nanozymes exhibits great potential in advancing photothermal–catalytic antibacterial therapy, thus emphasizing their promising role in future biomedical applications.

### 4.5. MOFs-Based Nanozymes

MOFs are crystalline materials composed of metal ions and organic bridging linkers, characterized by diverse porous structures and exhibiting attributes of exceptionally high porosity (up to 90% free volume) and a significant surface area within the organic framework [207]. Due to their porous structures, MOFs have gained significant attention as protective matrices for immobilizing enzymes through chemical grafting or physical adsorption, effectively preventing denaturation caused by external stimuli [208,209]. Liu et al. proposed a novel approach combining cerium complexes (DNase mimics) and MOFs with peroxidase-like activity to create an artificial nanozyme with dual enzyme-mimetic capabilities. This integrated nanozyme effectively disperses biofilms and eliminates bacteria without the adverse effects commonly associated with antibiotics. The cerium (IV) complexes acted as DNase mimics, effectively breaking down eDNA and disrupting mature biofilms. Meanwhile, the MOF with peroxidase-like activity killed bacteria in dispersed biofilms by using H_2_O_2_. The combination of these two types of nanozymes effectively prevented bacterial recolonization and biofilm reoccurrence, which is a rational strategy for combating biofilms considering the challenges associated with using single-modal antibacterial agents [166]. Hu et al. successfully synthesized ultrasmall gold nanoparticles (UsAuNPs) on ultrathin two-dimensional MOFs through an in situ reduction process. The resulting hybrid material, UsAuNPs/MOFs, combines the advantages of both UsAuNPs and ultrathin 2D MOFs. This hybrid material shows remarkable peroxidase-like activity, efficiently decomposing H_2_O_2_ into •OH. The antibacterial efficacy of the UsAuNPs/MOFs nanozyme was evaluated against both Escherichia coli (Gram-negative) and Staphylococcus aureus (Gram-positive), achieving excellent antibacterial properties with low concentrations of H_2_O_2_. Animal experiments showed that the hybrid material effectively promotes wound healing and is biocompatible, indicating the potential of hybrid nanozymes in antibacterial therapy for future clinical applications [210]. These innovative methodologies highlight the potential of MOFs and nanozymes in tackling the challenge of biofilm eradication. By leveraging the unique characteristics and synergistic interplay of these materials, researchers are striving to advance strategies that offer enhanced efficacy in combating biofilms. This is particularly crucial, as biofilms represent a persistent obstacle that is notoriously resistant to eradication using conventional monotherapy antibacterial agents.

Additionally, the integration of MOFs with other nanomaterials can manifest supplementary functional attributes. Through the amalgamation of MOFs and diverse materials, the resultant composite systems can exhibit synergistic effects, thereby augmenting antibacterial activity and efficacy [211]. In addition, by incorporating antibacterial agents into MOFs or utilizing MOFs as carriers for other materials, the controlled release of antimicrobial agents can be achieved, thereby ensuring prolonged and effective antibacterial activity [212]. Moreover, the porous structures of MOFs can function as protective matrices for immobilizing anti-bacterial agents, thereby preventing their degradation or premature release. This safeguarding mechanism contributes to the preservation of stability and efficacy of antibacterial agents, ultimately enhancing their durability and performance in antibacterial applications [213]. The combination of MOFs with other materials in antibacterial applications can provide synergistic antimicrobial properties, controlled release of antibacterial agents, and preservation of their efficacy.

## 5. Summary and Outlook

This review presents a comprehensive overview of recent advances in the utilization of nanozyme-based platforms for pathogen detection and control. Compared to their natural enzyme counterparts, nanozymes offer distinct advantages such as cost effectiveness, high stability, controllable size, ease of preparation, and superior catalytic activity. These features position nanozymes as highly promising agents for antibacterial applications and versatile detection media. Despite the remarkable performance of current nanozymes in detection and antibacterial activities, their application remains largely in the preliminary stage. The field of nanozymes for clinical antibacterial therapy and diagnoses has not made significant progress due to limited research efforts. Furthermore, the development of nanozymes still faces numerous challenges that must be addressed for further advancement and widespread implementation.

Primarily, it should be noted that the catalytic activity demonstrated by nanozymes is comparatively inferior to that of natural enzymes, thereby limiting their capacity to fully meet the demands of pathogen detection and control. Furthermore, recent investigations have confirmed that the catalytic performance of nanozymes is profoundly influenced by a multitude of intrinsic physicochemical properties such as shape, size, and surface modification, as well as extrinsic factors including temperature, pH, and substrate concentration. However, the understanding of how complex biological microenvironments impact the catalytic activity of nanozymes and their long-term effects remains largely unexplored.

Secondly, it is noteworthy that certain types of nanozymes exhibit limited substrate selectivity, which may lead to potential undesired effects in biological environments. Therefore, precise therapy based on nanozymes has emerged as a prominent research area requiring substantial efforts to enhance the selectivity of nanozymes for targeted therapeutic interventions. Building upon existing research, we envision that the achievement of substrate-specific identification and catalysis can be realized through the implementation of appropriate adaptations, such as incorporating aptamers, chiral molecules, or molecularly imprinted polymers onto the surface of nanozymes. Alternatively, the synergistic combination of nanozymes with natural enzymes possessing inherent substrate selectivity offers an alternative approach to overcome this limitation and advance targeted therapy applications.

Thirdly, it is worth noting that nanozymes are based on enzymatic principles; however, their properties significantly differ from those of natural enzymes. Therefore, conventional methods used to characterize enzyme performance face challenges when applied to nanozymes. The widely used Michaelis–Menten mechanism for analyzing natural enzymes is based on homogeneous catalysis in a uniform medium, while nanozymes operate through a heterogeneous mechanism that occurs on the surface of nanomaterials. Therefore, it is imperative to establish a consistent framework and standardized criteria for effectively evaluating and elucidating the performance attributes of nanozymes.

Fourthly, it is worth noting that current research endeavors pertaining to the detection of target analytes in food samples predominantly concentrate on liquid matrices, such as milk and juice beverages. Conversely, relatively inadequate attention has been directed towards the examination of solid food samples. Consequently, it is fundamental to allocate additional resources and conduct extensive investigations to further elucidate and explore this area of study.

Moreover, it is worth noting that a significant number of nanozymes designed for antibacterial applications exhibit notable inhibitory effects on bacterial growth in vitro. However, the translation of such findings into in vivo models and treatment strategies remains at an early stage. Considering the intricate nature of biological systems, intensifying in vivo investigations of nanozymes is advantageous for evaluating their therapeutic efficacy, biocompatibility, and biodegradability.

Lastly, despite the existence of several investigations proposing potential catalytic mechanisms of nanozymes, numerous challenges and obstacles persist in the comprehensive elucidation of their catalytic pathways. In forthcoming research endeavors, it is imperative to delve deeper into the catalytic reactions of nanozymes. The advancements achieved in the field of computer science, particularly in molecular dynamics’ simulations, offer promising opportunities for unraveling the intricate interplay between the structure, activity, and catalytic mechanisms exhibited by nanozymes. Accordingly, the utilization of collaborative computational simulations, theoretical calculations, and the integration of artificial intelligence techniques possesses significant potential in facilitating a comprehensive comprehension of the molecular-level catalytic mechanisms employed by nanozymes.

In conclusion, the field of nanozymes utilized in pathogen detection and control has witnessed significant advancements. However, it is crucial to acknowledge that current developments have not fully satisfied the requirements of practical applications thus far. Consequently, concerted efforts are imperative in addressing the unresolved challenges, which will contribute to substantial progress in forthcoming research endeavors. This comprehensive review is anticipated not only to enhance researchers’ engagement and enthusiasm in the field of nanozymes but also to provide invaluable information and insights that facilitate exploration of previously uncharted catalytic mechanisms inherent within nanozymes. Therefore, the aforementioned challenges represent the forefront of future nanozyme research, necessitating further exploration and investigation.

## Figures and Tables

**Table 1:** Mechanisms of the antibacterial activities of nanozymes by ROS regulation.

Nanozymes	Enzyme Mimicked	Bacterial Types	Antibacterial Mechanism	References
CeO_2_ nanozyme	Peroxidase	*E. coli*	Generation of •OH	[144]
Fe-N-C single-atom nanozyme	Peroxidase	*E. coli, S. aureus*	Generation of •OH	[23]
hybrid Ag/CeO_2_ nanocomposite	Superoxide dismutase	*S. aureus*	Generation of •O2	[145]
graphdiyne-nanowalls-wrapped hollow copper sulfide nanocubes (CuS@GDY)	Photothermal activity	*E. coli, methicillin-resistant S. aureus, S. aureus*	Generation of •OH and •O2	[147]
zinc oxide nanorod@graphdiyne nanosheet (ZnO@GDY NR)	Peroxidase	*methicillin-resistant* *S. aureus, P. aeruginosa*	Generation of •OH and •O2	[153]
Au@CeO_2_ hybrid nanozyme	Peroxidase	*E. coli, S. aureus*	Generation of •OH	[148]
TA-chelated Ag (TA-Ag) nanozyme	Peroxidase	*E. coli, S. epidermidis*	Generation of •OH	[154]
Mo@ZIF-8 nanozyme	Peroxidase	*E. coli, S. aureus*	Generation of •OH	[151]
HMPB@Cur@PDA nanozyme	Superoxide dismutase	*-*	Generation of •OH and •O2	[155]
Co^II^TBPP(bpy) nanozyme	Peroxidase	*P. aeruginosa, E. coli*, *B. amyloliquefaciens*, and *S. aureus*	Generation of •OH	[150]

**Table 2:** Mechanisms of the antibacterial activities of nanozymes by HOBr/Cl generation.

Nanozymes	Enzyme Mimicked	Bacterial Types	Antibacterial Mechanism	References
Vanadium pentoxide nanoparticles	Haloperoxidase	*E. coli, S. aureus*	Generation of HOBr	[158]
Cobalt-doped MoS_2_ (Co−MoS_2_)	Haloperoxidase	*E. coli, S. aureus*	Generation of HOBr	[161]
CeO_2−x_ NRs	Haloperoxidase	-	Generation of HOBr	[160]
CeO_2−x_	Haloperoxidase	*-*	Generation of HOBr	[156]

**Table 3:** Mechanisms of the antibacterial activities of nanozymes by extracellular DNA clearance.

Nanozymes	Enzyme Mimicked	Bacterial Types	Antibacterial Mechanism	References
DNase-AuNCs	DNase	-	NIR-activated PTT and PDT	[165]
CS@Fe/CDs	DNase and peroxidase	*E. coli, S. aureus*	Generation of •OH and cleavage of extracellular DNA	[168]
Au-Pt nanoparticles	Oxidation	*E. coli O157:H7*	Absorb DNA, photothermal conversion under NIR irradiation	[167]
Ag-Fe_3_O_4_@MoS_2_ MNPs nanocomposite	DNase	*S. mutans, E. faecalis*	Sustained release of Vanc, magnetic force exposure augments the penetration of MNPs through the various layers of biofilm	[169]
Cu_x_O-PDA	Peroxidase	*E. coli, S. aureus*	DNA degradation, lipid peroxidation, and biofilm eradication	[170]
MOF/Ce-based nanozymes	DNase and peroxidase	*S. aureus*	Generation of •OH, Ce^IV^ target and hydrolyze eDNA	[166]

## Data Availability

The data presented in this study are available in the article.

## References

[1] Al-Anazi K.A., Al-Jasser A.M. (2014). Infections Caused by Acinetobacter Baumannii in Recipients of Hematopoietic Stem Cell Transplantation. Front. Oncol..

[2] Cattoir V., Felden B. (2019). Future Antibacterial Strategies: From Basic Concepts to Clinical Challenges. J. Infect. Dis..

[3] Weinstein R.A., Gaynes R., Edwards J.R. (2005). National Nosocomial Infections Surveillance System Overview of Nosocomial Infections Caused by Gram-Negative Bacilli. Clin. Infect. Dis..

[4] Ding X., Wang A., Tong W., Xu F. (2019). Biodegradable Antibacterial Polymeric Nanosystems: A New Hope to Cope with Multidrug-Resistant Bacteria. Small.

[5] Wu Y., Song Z., Wang H., Han H. (2019). Endogenous Stimulus-Powered Antibiotic Release from Nanoreactors for a Combination Therapy of Bacterial Infections. Nat. Commun..

[6] Ibrahim M., Ahmad F., Yaqub B., Ramzan A., Imran A., Afzaal M., Mirza S.A., Mazhar I., Younus M., Akram Q. (2020). Current Trends of Antimicrobials Used in Food Animals and Aquaculture. Antibiotics and Antimicrobial Resistance Genes in the Environment.

[7] Chebotar I.V., Bocharova Y.A., Gur’ev A.S., Mayansky N.A. (2020). Bacteria Survival Strategies in Contact with Antibiotics. RCLD.

[8] (2022). Development of Bacterial Resistance to Antibiotics. JAMA.

[9] Li T., Wang Z., Guo J., de la Fuente-Nunez C., Wang J., Han B., Tao H., Liu J., Wang X. (2023). Bacterial Resistance to Antibacterial Agents: Mechanisms, Control Strategies, and Implications for Global Health. Sci. Total Environ..

[10] Robotham J., Graves N., Cookson B., Barnett A., Wilson J., Edgeworth J., Batra R., Cuthbertson B., Cooper B. (2011). Screening, Isolation, and Decolonisation Strategies in the Control of Meticillin Resistant Staphylococcus Aureus in Intensive Care Units: Cost Effectiveness Evaluation. BMJ-Br. Med. J..

[11] Silbert S., Kubasek C., Uy D., Widen R. (2014). Comparison of ESwab with Traditional Swabs for Detection of Methicillin-Resistant Staphylococcus Aureus Using Two Different Walk-Away Commercial Real-Time PCR Methods. J. Clin. Microbiol..

[12] Faron M., Buchan B., Vismara C., Lacchini C., Bielli A., Gesu G., Liebregts T., van Bree A., Jansz A., Soucy G. (2016). Automated Scoring of Chromogenic Media for Detection of Methicillin-Resistant Staphylococcus Aureus by Use of WASPLab Image Analysis Software. J. Clin. Microbiol..

[13] Malhotra-Kumar S., Haccuria K., MichielS M., Leven M., Poyart C., Hryniewicz W., Goossens H. (2008). MOSAR WP2 Study Team Current Trends in Rapid Diagnostics for Methicillin-Resistant Staphylococcus Aureus and Glycopeptide-Resistant Enterococcus Species. J. Clin. Microbiol..

[14] Nijhuis R., van Maarseveen N., van Hannen E., van Zwet A., Mascini E. (2014). A Rapid and High-Throughput Screening Approach for Methicillin-Resistant Staphylococcus Aureus Based on the Combination of Two Different Real-Time PCR Assays. J. Clin. Microbiol..

[15] Toleman M., Reuter S., Coll F., Harrison E., Blane B., Brown N., Torok M., Parkhill J., Peacock S. (2016). Systematic Surveillance Detects Multiple Silent Introductions and Household Transmission of Methicillin-Resistant Staphylococcus Aureus USA300 in the East of England. J. Infect. Dis..

[16] Poli M., Rivera V., Neal D. (2002). Sensitive and Specific Colorimetric ELISAs for Staphylococcus Aureus Enterotoxins A and B in Urine and Buffer. Toxicon.

[17] Cho I., Irudayaraj J. (2013). In-Situ Immuno-Gold Nanoparticle Network ELISA Biosensors for Pathogen Detection. Int. J. Food Microbiol..

[18] (2016). Hu Jinqiang; Lei Junting; Bai Yanhong; Wei Xiangke; Jing Jianzhou; Gao Hui; Sun Xincheng; Dong Caiwen; Geng Yao; Jiang Chunpeng Establishment of PCR-ELISA Technology for Staphylococcus Aureus in Food. Sci. Technol. Food Ind..

[19] Li Z., Hu J., Zhan Y., Shao Z., Gao M., Yao Q., Li Z., Sun S., Wang L. (2023). Coupling Bifunctional Nanozyme-Mediated Catalytic Signal Amplification and Label-Free SERS with Immunoassays for Ultrasensitive Detection of Pathogens in Milk Samples. Anal. Chem..

[20] Zhang L., Chen Y., Cheng N., Xu Y., Huang K., Luo Y., Wang P., Duan D., Xu W. (2017). Ultrasensitive Detection of Viable *Enterobacter Sakazakii* by a Continual Cascade Nanozyme Biosensor. Anal. Chem..

[21] Zhang Q., Wang X., Kang Y., Sun H., Liang Y., Liu J., Su Z., Dan J., Luo L., Yue T. (2021). Natural Products Self-Assembled Nanozyme for Cascade Detection of Glucose and Bacterial Viability in Food. Foods.

[22] Wu L., Wang X., Wu X., Xu S., Liu M., Cao X., Tang T., Huang X., Huang H. (2022). MnO_2_ Nanozyme-Mediated CRISPR-Cas12a System for the Detection of SARS-CoV-2. ACS Appl. Mater. Inter..

[23] Feng Y., Qin J., Zhou Y., Yue Q., Wei J. (2022). Spherical Mesoporous Fe-N-C Single-Atom Nanozyme for Photothermal and Catalytic Synergistic Antibacterial Therapy. J. Colloid. Interf. Sci..

[24] Maddheshiya S., Nara S. (2022). Recent Trends in Composite Nanozymes and Their Pro-Oxidative Role in Therapeutics. Front. Bioeng. Biotech..

[25] Xi J., Wei G., Wu Q., Xu Z., Liu Y., Han J., Fan L., Gao L. (2019). Light-Enhanced Sponge-like Carbon Nanozyme Used for Synergetic Antibacterial Therapy. Biomater. Sci..

[26] Zhang L., Qi Z., Zou Y., Zhang J., Xia W., Zhang R., He Z., Cai X., Lin Y., Duan S.-Z. (2019). Engineering DNA–Nanozyme Interfaces for Rapid Detection of Dental Bacteria. ACS Appl. Mater. Inter..

[27] Nicklen F.D., Diaz A.J., Lu J., Patel S.T., Zheng E.M., Campbell V.R., Wu B.M., Kamei D.T. (2022). Application of the Aqueous Two-Phase System and Nanozyme Signal Enhancement for the Improved Detection of Plasmodium Lactate Dehydrogenase in Serum. Anal. Bioanal. Chem..

[28] Liu X., Gao Y., Chandrawati R., Hosta-Rigau L. (2019). Therapeutic Applications of Multifunctional Nanozymes. Nanoscale.

[29] Sun C., Wang W., Sun X., Chu W., Yang J., Dai J., Ju Y. (2021). An Intrinsically Thermogenic Nanozyme for Synergistic Antibacterial Therapy. Biomater. Sci..

[30] Stasyuk N., Smutok O., Demkiv O., Prokopiv T., Gayda G., Nisnevitch M., Gonchar M. (2020). Synthesis, Catalytic Properties and Application in Biosensorics of Nanozymes and Electronanocatalysts: A Review. Sensors.

[31] Sun H., Cai S., Wang C., Chen Y., Yang R. (2020). Recent Progress of Nanozymes in the Detection of Pathogenic Microorganisms. ChemBioChem.

[32] Gao L., Zhuang J., Nie L., Zhang J., Zhang Y., Gu N., Wang T., Feng J., Yang D., Perrett S. (2007). Intrinsic Peroxidase-like Activity of Ferromagnetic Nanoparticles. Nat. Nanotechnol..

[33] He Y., Li X., Xu X., Pan J., Niu X. (2018). A Cobalt-Based Polyoxometalate Nanozyme with High Peroxidase-Mimicking Activity at Neutral PH for One-Pot Colorimetric Analysis of Glucose. J. Mater. Chem. B.

[34] Jiao L., Yan H.Y., Wu Y., Gu W.L., Zhu C.Z., Du D., Lin Y.H. (2020). When Nanozymes Meet Single-Atom Catalysis. Angew. Chem. Int. Ed..

[35] Jin X., Chen L., Zhang Y., Wang X., Zhou N. (2021). A Lateral Flow Strip for On-Site Detection of Tobramycin Based on Dual-Functional Platinum-Decorated Gold Nanoparticles. Analyst.

[36] Liu Q., Cao S., Sun Q., Xing C., Gao W., Lu X., Li X., Yang G., Yu S., Chen Y. (2022). A Perylenediimide Modified SiO_2_@TiO_2_ Yolk-Shell Light-Responsive Nanozyme: Improved Peroxidase-like Activity for H_2_O_2_ and Sarcosine Sensing. J. Hazard. Mater..

[37] Mei L., Zhu S., Liu Y., Yin W., Gu Z., Zhao Y. (2021). An Overview of the Use of Nanozymes in Antibacterial Applications. Chem. Eng. J..

[38] Huang Y., Mu X., Wang J., Wang Y., Xie J., Ying R., Su E. (2022). The Recent Development of Nanozymes for Food Quality and Safety Detection. J. Mater. Chem. B.

[39] Nguyen Q.H., Kim M.I. (2020). Nanomaterial-Mediated Paper-Based Biosensors for Colorimetric Pathogen Detection. TrAC Trends Anal. Chem..

[40] Songca S.P. (2022). Applications of Nanozymology in the Detection and Identification of Viral, Bacterial and Fungal Pathogens. Int. J. Mol. Sci..

[41] Feng F., Yang S., Ling Y., Jiang G.-B., Chu X.-G. (2012). Simultaneous Screening of 14 Illegal Food Additives in Wines Using Ultra Performance Liquid Chromatography Tandem Mass Spectrometry: Simultaneous Screening of 14 Illegal Food Additives in Wines Using Ultra Performance Liquid Chromatography Tandem Mass Spectrometry. Chin. J. Anal. Chem..

[42] Fu Y., Zhao C., Lu X., Xu G. (2017). Nontargeted Screening of Chemical Contaminants and Illegal Additives in Food Based on Liquid Chromatography–High Resolution Mass Spectrometry. TrAC Trends Anal. Chem..

[43] Huang L., Sun D., Pu H., Wei Q. (2019). Development of Nanozymes for Food Quality and Safety Detection: Principles and Recent Applications. Compr. Rev. Food Sci. Food Saf..

[44] Salarizadeh N., Sadri M., Sajedi R.H. (2018). Synthesis and Catalytic Evaluation of Fe_3_O_4_/MWCNTs Nanozyme as Recyclable Peroxidase Mimetics: Biochemical and Physicochemical Characterization. Appl. Organomet. Chem..

[45] Payal A., Krishnamoorthy S., Elumalai A., Moses J.A., Anandharamakrishnan C. (2021). A Review on Recent Developments and Applications of Nanozymes in Food Safety and Quality Analysis. Food Anal. Method..

[46] Wang W., Gunasekaran S. (2020). Nanozymes-Based Biosensors for Food Quality and Safety. TrAC Trends Anal. Chem..

[47] Takeuchi T. (2015). Molecular Imprinting for Proteins and Related Biomolecules—Preface. Mol. Imprinting.

[48] Li Z., Feng K., Zhang W., Ma M., Gu N., Zhang Y. (2018). Catalytic mechanism and application of nanozymes (in Chinese). Chin. Sci. Bull..

[49] Perez-Benito J.F. (2004). Iron(III)−Hydrogen Peroxide Reaction: Kinetic Evidence of a Hydroxyl-Mediated Chain Mechanism. J. Phys. Chem. A.

[50] Singh M., Rai V.K., Rai A., Graphene-Based Nanomaterials as Organocatalyst (2022). Graphene-Based Nanomaterial Catalysis.

[51] Song J., He J., Yang L., Wang W., Bai Q., Feng W., Li R. (2023). Enhanced Peroxidase-Like and Antibacterial Activity of Ir-CoatedPd-Pt Nanodendrites as Nanozyme. Bioinorg. Chem. Appl..

[52] Tian Y., Chen Y., Chen M., Song Z.-L., Xiong B., Zhang X.-B. (2021). Peroxidase-like Au@Pt Nanozyme as an Integrated Nanosensor for Ag+ Detection by LSPR Spectroscopy. Talanta.

[53] Gao L.-Z., Yan X.-Y. (2013). Discovery and Current Application of Nanozyme. Acta Agron. Sin..

[54] Bu S., Wang K., Wang C., Li Z., Hao Z., Liu W., Wan J. (2020). Immunoassay for Foodborne Pathogenic Bacteria Using Magnetic Composites Ab@Fe_3_O_4_, Signal Composites Ap@PtNp, and Thermometer Readings. Microchim. Acta.

[55] Das R., Chaterjee B., Kapil A., Sharma T.K. (2020). Aptamer-NanoZyme Mediated Sensing Platform for the Rapid Detection of *Escherichia Coli* in Fruit Juice. Sens. Bio-Sens. Res..

[56] Wang Z., Yao X., Zhang Y., Wang R., Ji Y., Sun J., Zhang D., Wang J. (2020). Functional Nanozyme Mediated Multi-Readout and Label-Free Lateral Flow Immunoassay for Rapid Detection of *Escherichia coli* O157:H7. Food Chem..

[57] Leng Y., Bu S., Li Z., Hao Z., Ma C., He X., Wan J. (2021). A Colorimetric Immunosensor Based on Hemin@MI Nanozyme Composites, with Peroxidase-like Activity for Point-of-Care Testing of Pathogenic *E. coli* O157:H7. Anal. Sci..

[58] Chen Y., Ma J., Yin X., Deng Z., Liu X., Yang D., Zhao L., Sun J., Wang J., Zhang D. (2023). Joint-Detection of Salmonella Typhimurium and *Escherichia Coli* O157:H7 by an Immersible Amplification Dip-Stick Immunoassay. Biosens. Bioelectron..

[59] Mu Z., Wu S., Guo J., Zhao M., Wang Y. (2022). Dual Mechanism Enhanced Peroxidase-like Activity of Iron–Nickel Bimetal–Organic Framework Nanozyme and Its Application for Biosensing. ACS Sustain. Chem. Eng..

[60] Zhou J., Tian F., Fu R., Yang Y., Jiao B., He Y. (2020). Enzyme–Nanozyme Cascade Reaction-Mediated Etching of Gold Nanorods for the Detection of *Escherichia coli*. ACS Appl. Nano Mater..

[61] Hong S.J., Chun H., Hong M., Han B. (2022). N- and B-Doped Fullerene as Peroxidase- and Catalase-Like Metal-Free Nanozymes with Ph-Switchable Catalytic Activity: A First-Principles Approach. SSRN J..

[62] Xu D., Wu L., Yao H., Zhao L. (2022). Catalase-Like Nanozymes: Classification, Catalytic Mechanisms, and Their Applications. Small.

[63] Zhou X., Fan C., Tian Q., Han C., Yin Z., Dong Z., Bi S. (2022). Trimetallic AuPtCo Nanopolyhedrons with Peroxidase- and Catalase-Like Catalytic Activity for Glow-Type Chemiluminescence Bioanalysis. Anal. Chem..

[64] Zhang X., Wu Y., Chen J., Yang Y., Li G. (2021). Bioinspired Artificial “Clickase” for the Catalytic Click Immunoassay of Foodborne Pathogens. Anal. Chem..

[65] Zhang X., Shi Y., Wang P., Wu D., Liu J., Huang R., Wu Y., Li G. (2023). Biomineralization-Inspired Artificial Clickase for Portable Click SERS Immunoassay of Salmonella Enterica Serovar Paratyphi B in Foods. Food Chem..

[66] Lang Y., Zhang B., Cai D., Tu W., Zhang J., Shentu X., Ye Z., Yu X. (2022). Determination Methods of the Risk Factors in Food Based on Nanozymes: A Review. Biosensors.

[67] Bockmann R. (2012). Internationale Koordinierung Nationaler Enforcement-Aktivitäten.

[68] Scallan E., Hoekstra R.M., Angulo F.J., Tauxe R.V., Widdowson M.-A., Roy S.L., Jones J.L., Griffin P.M. (2011). Foodborne Illness Acquired in the United States—Major Pathogens. Emerg. Infect. Dis..

[69] Matle I., Mbatha K.R., Madoroba E. (2020). A Review of Listeria Monocytogenes from Meat and Meat Products: Epidemiology, Virulence Factors, Antimicrobial Resistance and Diagnosis. Onderstepoort J. Vet..

[70] Shi D., Shi H. (2022). Combining Loop-Mediated Isothermal Amplification and Nanozyme-Strip for Ultrasensitive and Rapid Detection of Viable Listeria Monocytogenes Cells and Biofilms. LWT.

[71] Wu Z., Huang C., Dong Y., Zhao B., Chen Y. (2022). Gold Core @ Platinum Shell Nanozyme-Mediated Magnetic Relaxation Switching DNA Sensor for the Detection of Listeria Monocytogenes in Chicken Samples. Food Control.

[72] Drinković D., Taylor S.L., Lang S. (2004). Five Cases of Non-Typhoidal *Salmonella* Endovascular Infection: Non-Typhoidal *Salmonella*. Intern. Med. J..

[73] Hu J., Tang F., Jiang Y.-Z., Liu C. (2020). Rapid Screening and Quantitative Detection of *Salmonella* Using a Quantum Dot Nanobead-Based Biosensor. Analyst.

[74] Jin L., Li T., Wu B., Yang T., Zou D., Liang X., Hu L., Huang G., Zhang J. (2020). Rapid Detection of Salmonella in Milk by Nuclear Magnetic Resonance Based on Membrane Filtration Superparamagnetic Nanobiosensor. Food Control.

[75] Guo R., Xue L., Cai G., Qi W., Liu Y., Lin J. (2021). Fe-MIL-88NH_2_ Metal–Organic Framework Nanocubes Decorated with Pt Nanoparticles for the Detection of *Salmonella*. ACS Appl. Nano Mater..

[76] Wang L., Zhou H., Hu H., Wu X., Guo W., Liu Y., Huang Y., Yang X., Chen X. (2022). Constructing Difunctional Histidine-Modified Magnetic Hybrid Nanozymes as Capture Probes and Signal Amplifiers for the Sensitive Colorimetric Detection of Salmonella Typhimurium in Food. Microchem. J..

[77] Kadariya J., Smith T.C., Thapaliya D. (2014). *Staphylococcus Aureus* and Staphylococcal Food-Borne Disease: An Ongoing Challenge in Public Health. BioMed Res. Int..

[78] Chakraborty S.P., Mahapatra S.K., Sahu S.K., Chattopadhyay S., Pramanik P., Roy S. (2011). Nitric Oxide Mediated Staphylococcus Aureus Pathogenesis and Protective Role of Nanoconjugated Vancomycin. Asian Pac. J. Trop. Bio..

[79] Wang X., Zhang M., Pang X., Huang K., Yao Z., Mei X., Cheng N. (2022). Comparative Study of Pd@Pt Nanozyme Improved Colorimetric N-ELISA for the Paper-Output Portable Detection of Staphylococcus Aureus. Talanta.

[80] Wei L., Wang Z., Wang J., Wang X., Chen Y. (2022). Aptamer-Based Colorimetric Detection of Methicillin-Resistant Staphylococcus Aureus by Using a CRISPR/Cas12a System and Recombinase Polymerase Amplification Br. Anal. Chim. Acta.

[81] Wellinghausen N., Siegel D., Gebert S., Winter J. (2009). Rapid Detection of Staphylococcus Aureus Bacteremia and Methicillin Resistance by Real-Time PCR in Whole Blood Samples. Eur. J. Clin. Microbiol..

[82] Hait J.M., Tallent S.M., Bennett R.W. (2014). Screening, Detection, and Serotyping Methods for Toxin Genes and Enterotoxins in *Staphylococcus* Strains. J. Aoac. Int..

[83] Lian Y., He F., Wang H., Tong F. (2015). A New Aptamer/Graphene Interdigitated Gold Electrode Piezoelectric Sensor for Rapid and Specific Detection of Staphylococcus Aureus. Biosens. Bioelectron..

[84] Bunka D.H.J., Stockley P.G. (2006). Aptamers Come of Age—At Last. Nat. Rev. Microbiol..

[85] Zhu S., Tang Y., Shi B., Zou W., Wang X., Wang C., Wu Y. (2021). Oligonucleotide-Mediated the Oxidase-Mimicking Activity of Mn3O4 Nanoparticles as a Novel Colorimetric Aptasensor for Ultrasensitive and Selective Detection of Staphylococcus Aureus in Food. Sens. Actuat B-Chem..

[86] Phetcharaburanin J., Deewai S., Kulthawatsiri T., Moolpia K., Suksawat M., Promraksa B., Klanrit P., Namwat N., Loilome W., Poopasit K. (2020). 1H NMR Metabolic Phenotyping of Dipterocarpus Alatus as a Novel Tool for Age and Growth Determination. PLoS ONE.

[87] Lin X., Ibarlucea B., Peng T., Shen R., Li P., Zhang P. (2023). Two Birds with One Stone: A Multifunctional Nanoplatform for Photothermal Sensitive Detection and Real-Time Inactivation of Staphylococcus Aureus with NIR Responsive Cu_2−X_Se@Van NPs. Sens. Actuat B-Chem..

[88] Wu S.-C., Tsai T.-T., Li T.-H., Tung C.-Y., Chiu P.-Y., Lin J.-H., Chen C.-F. (2022). Palladium-Platinum Bimetallic Nanomaterials and Their Application in Staphylococcus Aureus Detection on Paper-Based Devices. Biosens. Bioelectron..

[89] Mohamad A., Teo H., Keasberry N.A., Ahmed M.U. (2019). Recent Developments in Colorimetric Immunoassays Using Nanozymes and Plasmonic Nanoparticles. Crit. Rev. Biotechnol..

[90] Jiao L., Yan H., Xu W., Wu Y., Gu W., Li H., Du D., Lin Y., Zhu C. (2019). Self-Assembly of All-Inclusive Allochroic Nanoparticles for the Improved ELISA. Anal. Chem..

[91] Wang S., Shen W., Zheng S., Li Z., Wang C., Zhang L., Liu Y. (2021). Dual-Signal Lateral Flow Assay Using Vancomycin-Modified Nanotags for Rapid and Sensitive Detection of *Staphylococcus Aureus*. Rsc. Adv..

[92] Wang J., Yang W., Peng Q., Han D., Kong L., Fan L., Zhao M., Ding S. (2020). Rapid Detection of Carbapenem-Resistant Enterobacteriaceae Using PH Response Based on Vancomycin-Modified Fe_3_O_4_ @Au Nanoparticle Enrichment and the Carbapenemase Hydrolysis Reaction. Anal. Methods-UK.

[93] Zhu S., Wu Z., Niu X., Zhan X., Tao H., Wu Y. (2022). Novel Nanozyme-Catalyzed and Magnetically Assisted Colorimetric Biosensor for Staphylococcus Aureus Detection with a Low Matrix Effect from Complex Environments. Sens. Actuat B-Chem..

[94] Leung L.M., Fondrie W.E., Doi Y., Johnson J.K., Strickland D.K., Ernst R.K., Goodlett D.R. (2017). Identification of the ESKAPE Pathogens by Mass Spectrometric Analysis of Microbial Membrane Glycolipids. Sci. Rep.-UK.

[95] Tang Y., Ali Z., Zou J., Jin G., Zhu J., Yang J., Dai J. (2017). Detection Methods for Pseudomonas Aeruginosa: History and Future Perspective. Rsc. Adv..

[96] Mena K.D., Gerba C.P., Whitacre D.M. (2009). Risk Assessment of Pseudomonas Aeruginosa in Water. Reviews of Environmental Contamination and Toxicology Vol 201.

[97] Alves-Balvedi R.P., Caetano L.P., Madurro J.M., Brito-Madurro A.G. (2016). Use of 3,3′,5,5′ Tetramethylbenzidine as New Electrochemical Indicator of DNA Hybridization and Its Application in Genossensor. Biosens. Bioelectron..

[98] Das R., Dhiman A., Kapil A., Bansal V., Sharma T.K. (2019). Aptamer-Mediated Colorimetric and Electrochemical Detection of Pseudomonas Aeruginosa Utilizing Peroxidase-Mimic Activity of Gold NanoZyme. Anal. Bioanal. Chem..

[99] Lee Y.J., Kim J., Jeon J.H., Seok H., Choi W.S., Chang E.-A., Yim H.J., Park D.W. (2021). Extraintestinal Manifestation of Yersinia Pseudotuberculosis Bacteremia as Acute Hepatitis: Case Report and Review of the Literature. Pathogens.

[100] Bonardi S., Bruini I., D’Incau M., Van Damme I., Carniel E., Brémont S., Cavallini P., Tagliabue S., Brindani F. (2016). Detection, Seroprevalence and Antimicrobial Resistance of Yersinia Enterocolitica and Yersinia Pseudotuberculosis in Pig Tonsils in Northern Italy. Int. J. Food Microbiol..

[101] Sundin C., Zetterström C.E., Vo D.D., Brkljača R., Urban S., Elofsson M. (2020). Exploring Resveratrol Dimers as Virulence Blocking Agents—Attenuation of Type III Secretion in Yersinia Pseudotuberculosis and Pseudomonas Aeruginosa. Sci. Rep-UK.

[102] Farooq U., Yang Q., Ullah M.W., Wang S. (2018). Bacterial Biosensing: Recent Advances in Phage-Based Bioassays and Biosensors. Biosens. Bioelectron..

[103] Yang Q., Wu D., Aziz A., Deng S., Zhou L., Chen W., Asif M., Wang S. (2023). Colorimetric Platform Based on Synergistic Effect between Bacteriophage and AuPt Nanozyme for Determination of Yersinia Pseudotuberculosis. Microchim. Acta.

[104] Brazaca L.C., Dos Santos P.L., De Oliveira P.R., Rocha D.P., Stefano J.S., Kalinke C., Abarza Muñoz R.A., Bonacin J.A., Janegitz B.C., Carrilho E. (2021). Biosensing Strategies for the Electrochemical Detection of Viruses and Viral Diseases—A Review. Anal. Chim. Acta.

[105] Sánchez-Báscones E., Parra F., Lobo-Castañón M.J. (2021). Aptamers against Viruses: Selection Strategies and Bioanalytical Applications. TrAC Trends Anal. Chem..

[106] Ebrahimi A., Alam M.A. (2015). Incubation-Free Detection of Bacteria Cells by Using Droplet-Based Impedance Sensing. Proceedings of the 2015 73rd Annual Device Research Conference (DRC).

[107] Birkenmeyer L.G. (2003). Hepatitis B Virus: Life Cycle and Morphogenesis. Perspectives in Medical Virology.

[108] Zhang S., Tian D., Zhang Z., Xiong J., Yuan Q., Ge S., Zhang J., Xia N. (2009). Clinical Significance of Anti-HEV IgA in Diagnosis of Acute Genotype 4 Hepatitis E Virus Infection Negative for Anti-HEV IgM. Dig. Dis. Sci..

[109] Khoris I.M., Chowdhury A.D., Li T.-C., Suzuki T., Park E.Y. (2020). Advancement of Capture Immunoassay for Real-Time Monitoring of Hepatitis E Virus-Infected Monkey. Anal. Chim. Acta.

[110] Parashar R.K. (2008). Reaction Mechanisms in Organic Synthesis: Parashar/Reaction Mechanisms in Organic Synthesis.

[111] Wang N., Pan G., Liu P., Rong S., Gao Z., Li Q. (2021). Advances and Future Perspective on Detection Technology of Human Norovirus. Pathogens.

[112] Weerathunge P., Ramanathan R., Torok V.A., Hodgson K., Xu Y., Goodacre R., Behera B.K., Bansal V. (2019). Ultrasensitive Colorimetric Detection of Murine Norovirus Using NanoZyme Aptasensor. Anal. Chem..

[113] Sharma R., Kakkar P. (2016). Software Module Fault Prediction Using Convolutional Neural Network with Feature Selection. IJSEIA.

[114] Bouthry E., Picone O., Hamdi G., Grangeot-Keros L., Ayoubi J.-M., Vauloup-Fellous C. (2014). Rubella and Pregnancy: Diagnosis, Management and Outcomes: Rubella and Pregnancy. Prenat. Diagn..

[115] Helfand R.F., Keyserling H.L., Williams I., Murray A., Mei J., Moscatiello C., Icenogle J., Bellini W.J. (2001). Comparative Detection of Measles and Rubella IgM and IgG Derived from Filter Paper Blood and Serum Samples. J. Med. Virol..

[116] Helfand R.F., Cabezas C., Abernathy E., Castillo-Solorzano C., Ortiz A.C., Sun H., Osores F., Oliveira L., Whittembury A., Charles M. (2007). Dried Blood Spots versus Sera for Detection of Rubella Virus-Specific Immunoglobulin M (IgM) and IgG in Samples Collected during a Rubella Outbreak in Peru. Clin. Vaccine Immunol..

[117] Raynal M., Ballester P., Vidal-Ferran A., Van Leeuwen P.W.N.M. (2014). Supramolecular Catalysis. Part 2. Artificial Enzyme Mimics. Chem. Soc. Rev..

[118] Li A., Long L., Liu F., Liu J., Wu X., Ji Y. (2019). Antigen-Labeled Mesoporous Silica-Coated Au-Core Pt-Shell Nanostructure: A Novel Nanoprobe for Highly Efficient Virus Diagnosis. J. Biol. Eng..

[119] Shi R., Shan C., Duan X., Chen Z., Liu P., Song J., Song T., Bi X., Han C., Wu L. (2020). A Human Neutralizing Antibody Targets the Receptor-Binding Site of SARS-CoV-2. Nature.

[120] (2023). The Lancet Hiv Opt-out HIV Testing in the UK. Lancet HIV.

[121] Bradbury D.W., Trinh J.T., Ryan M.J., Cantu C.M., Lu J., Nicklen F.D., Du Y., Sun R., Wu B.M., Kamei D.T. (2021). On-Demand Nanozyme Signal Enhancement at the Push of a Button for the Improved Detection of SARS-CoV-2 Nucleocapsid Protein in Serum. Analyst.

[122] Liu B., Wu Z., Liang C., Lu J., Li J., Zhang L., Li T., Zhao W., Fu Y., Hou S. (2021). Development of a Smartphone-Based Nanozyme-Linked Immunosorbent Assay for Quantitative Detection of SARS-CoV-2 Nucleocapsid Phosphoprotein in Blood. Front. Microbiol..

[123] Oh S., Kim J., Tran V.T., Lee D.K., Ahmed S.R., Hong J.C., Lee J., Park E.Y., Lee J. (2018). Magnetic Nanozyme-Linked Immunosorbent Assay for Ultrasensitive Influenza A Virus Detection. ACS Appl. Mater. Inter..

[124] Ding X., Yin K., Li Z., Liu C. (2020). All-in-One Dual CRISPR-Cas12a (AIOD-CRISPR) Assay: A Case for Rapid, Ultrasensitive and Visual Detection of Novel Coronavirus SARS-CoV-2 and HIV Virus. Res. Sq..

[125] Lucia C., Federico P.-B., Alejandra G.C. (2020). An Ultrasensitive, Rapid, and Portable Coronavirus SARS-CoV-2 Sequence Detection Method Based on CRISPR-Cas12. bioRxiv.

[126] Sridhara S., Goswami H.N., Whyms C., Dennis J.H., Li H. (2021). Virus Detection via Programmable Type III-A CRISPR-Cas Systems. Nat. Commun..

[127] Schwinn M.K., Machleidt T., Zimmerman K., Eggers C.T., Dixon A.S., Hurst R., Hall M.P., Encell L.P., Binkowski B.F., Wood K.V. (2018). CRISPR-Mediated Tagging of Endogenous Proteins with a Luminescent Peptide. ACS Chem. Biol..

[128] Chen Q., Tian T., Xiong E., Wang P., Zhou X. (2019). CRISPR/Cas13a Signal Amplification Linked Immunosorbent Assay (CLISA). bioRxiv.

[129] Mimitou E.P., Cheng A., Montalbano A., Hao S., Stoeckius M., Legut M., Roush T., Herrera A., Papalexi E., Ouyang Z. (2019). Multiplexed Detection of Proteins, Transcriptomes, Clonotypes and CRISPR Perturbations in Single Cells. Nat. Methods.

[130] Liang M., Li Z., Wang W., Liu J., Liu L., Zhu G., Karthik L., Wang M., Wang K.-F., Wang Z. (2019). A CRISPR-Cas12a-Derived Biosensing Platform for the Highly Sensitive Detection of Diverse Small Molecules. Nat. Commun..

[131] Xiong Y., Zhang J., Yang Z., Mou Q., Ma Y., Xiong Y., Lu Y. (2020). Functional DNA Regulated CRISPR-Cas12a Sensors for Point-of-Care Diagnostics of Non-Nucleic-Acid Targets. J. Am. Chem. Soc..

[132] Niu C., Wang C., Li F., Zheng X., Xing X., Zhang C. (2021). Aptamer Assisted CRISPR-Cas12a Strategy for Small Molecule Diagnostics. Biosens. Bioelectron..

[133] Waggoner J.J., Gresh L., Mohamed-Hadley A., Ballesteros G., Davila M.J.V., Tellez Y., Sahoo M.K., Balmaseda A., Harris E., Pinsky B.A. (2016). Single-Reaction Multiplex Reverse Transcription PCR for Detection of Zika, Chikungunya, and Dengue Viruses. Emerg. Infect. Dis..

[134] Waggoner J.J., Pinsky B.A. (2016). Zika Virus: Diagnostics for an Emerging Pandemic Threat. J. Clin. Microbiol..

[135] Johansson M.A., Mier-y-Teran-Romero L., Reefhuis J., Gilboa S.M., Hills S.L. (2016). Zika and the Risk of Microcephaly. N. Engl. J. Med..

[136] Cauchemez S., Besnard M., Bompard P., Dub T., Guillemette-Artur P., Eyrolle-Guignot D., Salje H., Van Kerkhove M.D., Abadie V., Garel C. (2016). Association between Zika Virus and Microcephaly in French Polynesia, 2013–2015: A Retrospective Study. Lancet.

[137] Hsu Y.-P., Li N.-S., Chen Y.-T., Pang H.-H., Wei K.-C., Yang H.-W. (2020). A Serological Point-of-Care Test for Zika Virus Detection and Infection Surveillance Using an Enzyme-Free Vial Immunosensor with a Smartphone. Biosens. Bioelectron..

[138] Fact Sheet about Malaria. https://www.who.int/news-room/fact-sheets/detail/malaria.

[139] Murray C.K., Bennett J.W. (2009). Rapid Diagnosis of Malaria. Interdiscip. Perspect. Infect. Dis..

[140] Zhang C., Wang X., Du J., Gu Z., Zhao Y. (2021). Reactive Oxygen Species-Regulating Strategies Based on Nanomaterials for Disease Treatment. Adv. Sci..

[141] Shadel G.S., Horvath T.L. (2015). Mitochondrial ROS Signaling in Organismal Homeostasis. Cell.

[142] Yang Z., Min Z., Yu B. (2020). Reactive Oxygen Species and Immune Regulation. Int. Rev. Immunol..

[143] (2019). Biomolecules Editorial Office Acknowledgement to Reviewers of Biomolecules in 2018. Biomolecules.

[144] Zhu W., Wang L., Li Q., Jiao L., Yu X., Gao X., Qiu H., Zhang Z., Bing W. (2021). Will the Bacteria Survive in the CeO_2_ Nanozyme-H2O2 System?. Molecules.

[145] Li Y., Li S., Zhou R., Li G., Li X. (2023). Selective Laser Welding in Liquid: A Strategy for Preparation of High-Antibacterial Activity Nanozyme against Staphylococcus Aureus. J. Adv. Res..

[146] Korsvik C., Patil S., Seal S., Self W.T. (2007). Superoxide Dismutase Mimetic Properties Exhibited by Vacancy Engineered Ceria Nanoparticles. Chem. Commun..

[147] Bai Q., Liang M., Wu W., Zhang C., Li X., Liu M., Yang D., Yu W.W., Hu Q., Wang L. (2022). Plasmonic Nanozyme of Graphdiyne Nanowalls Wrapped Hollow Copper Sulfide Nanocubes for Rapid Bacteria-Killing. Adv. Funct. Mater..

[148] Liu C., Zhang M., Geng H., Zhang P., Zheng Z., Zhou Y., He W. (2021). NIR Enhanced Peroxidase-like Activity of Au@CeO_2_ Hybrid Nanozyme by Plasmon-Induced Hot Electrons and Photothermal Effect for Bacteria Killing. Appl. Catal. B-Env..

[149] Chen J., Xu F., Zhang Q., Li S. (2021). N-Doped MoS_2_-Nanoflowers as Peroxidase-like Nanozymes for Total Antioxidant Capacity Assay. Anal. Chim. Acta.

[150] Hu Y., Wang W., Huang S., Li J., Zhang Y., Gao Y., Cheng Y., Wu Z., Zhang X. (2022). A Targeted Nanozyme Based on Multiple Porphyrins for Enhanced Photodynamic Antibacterial Application. Chem. Eng. J..

[151] Lian Z., Lu C., Zhu J., Zhang X., Wu T., Xiong Y., Sun Z., Yang R. (2022). Mo@ZIF-8 Nanozyme Preparation and Its Antibacterial Property Evaluation. Front. Chem..

[152] Yang B., Chen Y., Shi J. (2019). Reactive Oxygen Species (ROS)-Based Nanomedicine. Chem. Rev..

[153] Bai Q., Zhang J., Yu Y., Zhang C., Jiang Y., Yang D., Liu M., Wang L., Du F., Sui N. (2022). Piezoelectric Activatable Nanozyme-Based Skin Patch for Rapid Wound Disinfection. ACS Appl. Mater. Inter..

[154] Jia Z., Lv X., Hou Y., Wang K., Ren F., Xu D., Wang Q., Fan K., Xie C., Lu X. (2021). Mussel-Inspired Nanozyme Catalyzed Conductive and Self-Setting Hydrogel for Adhesive and Antibacterial Bioelectronics. Bioact. Mater..

[155] Da J., Li Y., Zhang K., Ren J., Wang J., Liu X., Liu X., Zhang J., Liu L., Zhang W. (2022). Functionalized Prussian Blue Nanozyme as Dual-Responsive Drug Therapeutic Nanoplatform Against Maxillofacial Infection via Macrophage Polarization. Int. J. Nanomed..

[156] Herget K., Hubach P., Pusch S., Deglmann P., Götz H., Gorelik T.E., Gural’skiy I.A., Pfitzner F., Link T., Schenk S. (2017). Haloperoxidase Mimicry by CeO_2−*x*_ Nanorods Combats Biofouling. Adv. Mater..

[157] Wang W., Luo Q., Li J., Li Y., Wu R., Li Y., Huo X., Wang N. (2022). Single-Atom Tungsten Engineering of MOFs with Biomimetic Antibiofilm Activity toward Robust Uranium Extraction from Seawater. Chem. Eng. J..

[158] Natalio F., André R., Hartog A.F., Stoll B., Jochum K.P., Wever R., Tremel W. (2012). Vanadium Pentoxide Nanoparticles Mimic Vanadium Haloperoxidases and Thwart Biofilm Formation. Nat. Nanotechnol..

[159] Wever R., Tromp M.G.M., Krenn B.E., Marjani A., Van Tol M. (1991). Brominating Activity of the Seaweed Ascophyllum Nodosum: Impact on the Biosphere. Env. Sci. Technol..

[160] Hu M., Korschelt K., Viel M., Wiesmann N., Kappl M., Brieger J., Landfester K., Thérien-Aubin H., Tremel W. (2018). Nanozymes in Nanofibrous Mats with Haloperoxidase-like Activity To Combat Biofouling. ACS Appl. Mater. Inter..

[161] Luo Q., Li J., Wang W., Li Y., Li Y., Huo X., Li J., Wang N. (2022). Transition Metal Engineering of Molybdenum Disulfide Nanozyme for Biomimicking Anti-Biofouling in Seawater. ACS Appl. Mater. Inter..

[162] Davies D. (2003). Understanding Biofilm Resistance to Antibacterial Agents. Nat. Rev. Drug Discov..

[163] Lemire J.A., Harrison J.J., Turner R.J. (2013). Antimicrobial Activity of Metals: Mechanisms, Molecular Targets and Applications. Nat. Rev. Microbiol..

[164] Gupta A., Das R., Yesilbag Tonga G., Mizuhara T., Rotello V.M. (2018). Charge-Switchable Nanozymes for Bioorthogonal Imaging of Biofilm-Associated Infections. ACS Nano.

[165] Xie Y., Zheng W., Jiang X. (2020). Near-Infrared Light-Activated Phototherapy by Gold Nanoclusters for Dispersing Biofilms. ACS Appl. Mater. Inter..

[166] Liu Z., Wang F., Ren J., Qu X. (2019). A Series of MOF/Ce-Based Nanozymes with Dual Enzyme-like Activity Disrupting Biofilms and Hindering Recolonization of Bacteria. Biomaterials.

[167] Lu C., Tang L., Gao F., Li Y., Liu J., Zheng J. (2021). DNA-Encoded Bimetallic Au-Pt Dumbbell Nanozyme for High-Performance Detection and Eradication of *Escherichia Coli* O157:H7. Biosens. Bioelectron..

[168] Pan T., Chen H., Gao X., Wu Z., Ye Y., Shen Y. (2022). Engineering Efficient Artificial Nanozyme Based on Chitosan Grafted Fe-Doped-Carbon Dots for Bacteria Biofilm Eradication. J. Hazard. Mater..

[169] Baig M.M.F.A., Fatima A., Gao X., Farid A., Ajmal Khan M., Zia A.W., Wu H. (2022). Disrupting Biofilm and Eradicating Bacteria by Ag-Fe3O4@MoS2 MNPs Nanocomposite Carrying Enzyme and Antibiotics. J. Control Release.

[170] He S., Feng Y., Sun Q., Xu Z., Zhang W. (2022). Charge-Switchable Cu*_x_*O Nanozyme with Peroxidase and Near-Infrared Light Enhanced Photothermal Activity for Wound Antibacterial Application. ACS Appl. Mater. Inter..

[171] Nong W., Chen Y., Lv D., Yan Y., Zheng X., Shi X., Xu Z., Guan W., Wu J., Guan Y. (2022). Metal-Organic Framework Based Nanozyme Hybrid for Synergistic Bacterial Eradication by Lysozyme and Light-Triggered Carvacrol Release. Chem. Eng. J..

[172] Chen Z., Wang Z., Ren J., Qu X. (2018). Enzyme Mimicry for Combating Bacteria and Biofilms. Acc. Chem. Res..

[173] Wei F., Cui X., Wang Z., Dong C., Li J., Han X. (2021). Recoverable Peroxidase-like Fe_3_O_4_@MoS_2_-Ag Nanozyme with Enhanced Antibacterial Ability. Chem. Eng. J..

[174] Durmus N.G., Taylor E.N., Kummer K.M., Webster T.J. (2013). Enhanced Efficacy of Superparamagnetic Iron Oxide Nanoparticles Against Antibiotic-Resistant Biofilms in the Presence of Metabolites. Adv. Mater..

[175] Taylor E.N., Kummer K.M., Durmus N.G., Leuba K., Tarquinio K.M., Webster T.J. (2012). Superparamagnetic Iron Oxide Nanoparticles (SPION) for the Treatment of Antibiotic-Resistant Biofilms. Small.

[176] Wang T., Bai Q., Zhu Z., Xiao H., Jiang F., Du F., Yu W.W., Liu M., Sui N. (2021). Graphdiyne-Supported Palladium-Iron Nanosheets: A Dual-Functional Peroxidase Mimetic Nanozyme for Glutathione Detection and Antibacterial Application. Chem. Eng. J..

[177] Jiang B., Fang L., Wu K., Yan X., Fan K. (2020). Ferritins as Natural and Artificial Nanozymes for Theranostics. Theranostics.

[178] Ali S.R., De M. (2022). Fe-Doped MoS_2_ Nanozyme for Antibacterial Activity and Detoxification of Mustard Gas Simulant. ACS Appl. Mater. Inter..

[179] Li Y., Sun J., Huang L., Liu S., Wang S., Zhang D., Zhu M., Wang J. (2022). Nanozyme-encoded Luminescent Detection for Food Safety Analysis: An Overview of Mechanisms and Recent Applications. Compr. Rev. Food Sci. Food Saf..

[180] Hao J., Zhang C., Feng C., Wang Q., Liu Z.-Y., Li Y., Mu J., Yang E.-C., Wang Y. (2023). An Ultra-Highly Active Nanozyme of Fe,N Co-Doped Ultrathin Hollow Carbon Framework for Antibacterial Application. Chin. Chem. Lett..

[181] Liao Z.-Y., Gao W.-W., Shao N.-N., Zuo J.-M., Wang T., Xu M.-Z., Zhang F.-X., Xia Y.-M. (2022). Iron Phosphate Nanozyme–Hydrogel with Multienzyme-like Activity for Efficient Bacterial Sterilization. ACS Appl. Mater. Inter..

[182] Shi S., Zhang Q., Sun H., Su Z., Dan J., Liang Y., Kang Y., Du T., Sun J., Wang J. (2022). Glucose Oxidase-Integrated Metal-Polyphenolic Network as a Microenvironment-Activated Cascade Nanozyme for Hyperglycemic Wound Disinfection. ACS Biomater. Sci. Eng..

[183] Huang W.-C., Lyu L.-M., Yang Y.-C., Huang M.H. (2012). Synthesis of Cu_2_O Nanocrystals from Cubic to Rhombic Dodecahedral Structures and Their Comparative Photocatalytic Activity. J. Am. Chem. Soc..

[184] Hu L., Yuan Y., Zhang L., Zhao J., Majeed S., Xu G. (2013). Copper Nanoclusters as Peroxidase Mimetics and Their Applications to H_2_O_2_ and Glucose Detection. Anal. Chim. Acta.

[185] Liang Y., Han Y., Dan J., Li R., Sun H., Wang J., Zhang W. (2023). A High-Efficient and Stable Artificial Superoxide Dismutase Based on Functionalized Melanin Nanoparticles from Cuttlefish Ink for Food Preservation. Food Res. Int..

[186] Liu Y., Nie N., Tang H., Zhang C., Chen K., Wang W., Liu J. (2021). Effective Antibacterial Activity of Degradable Copper-Doped Phosphate-Based Glass Nanozymes. ACS Appl. Mater. Inter..

[187] Li H.-W., Mao J.-Y., Lien C.-W., Wang C.-K., Lai J.-Y., Mandal R.P., Chang H.-T., Chang L., Hui-Kang D., Huang C.-C. (2020). Platinum Ions Mediate the Interactions between DNA and Carbon Quantum Dots: Diagnosis of MRSA Infections. J. Mater. Chem. B.

[188] Sun D., Pang X., Cheng Y., Ming J., Xiang S., Zhang C., Lv P., Chu C., Chen X., Liu G. (2020). Ultrasound-Switchable Nanozyme Augments Sonodynamic Therapy against Multidrug-Resistant Bacterial Infection. ACS Nano.

[189] Wang J., Mu X., Li Y., Xu F., Long W., Yang J., Bian P., Chen J., Ouyang L., Liu H. (2018). Hollow PtPdRh Nanocubes with Enhanced Catalytic Activities for In Vivo Clearance of Radiation-Induced ROS via Surface-Mediated Bond Breaking. Small.

[190] Mao Y., Jia F., Jing T., Li T., Jia H., He W. (2021). Enhanced Multiple Enzymelike Activity of PtPdCu Trimetallic Nanostructures for Detection of Fe^2+^ and Evaluation of Antioxidant Capability. ACS Sustain. Chem. Eng..

[191] Bligaard T., Nørskov J.K., Dahl S., Matthiesen J., Christensen C.H., Sehested J. (2004). The Brønsted–Evans–Polanyi Relation and the Volcano Curve in Heterogeneous Catalysis. J. Catal..

[192] Kari J., Olsen J.P., Jensen K., Badino S.F., Krogh K.B.R.M., Borch K., Westh P. (2018). Sabatier Principle for Interfacial (Heterogeneous) Enzyme Catalysis. ACS Catal..

[193] Fang G., Li W., Shen X., Perez-Aguilar J.M., Chong Y., Gao X., Chai Z., Chen C., Ge C., Zhou R. (2018). Differential Pd-Nanocrystal Facets Demonstrate Distinct Antibacterial Activity against Gram-Positive and Gram-Negative Bacteria. Nat. Commun..

[194] Yang D., Li Q., Zhang Q., Wang Y., Li H., Tammina S.K., Yang Y. (2022). A Multifunctional Nanozyme-Based Enhanced System for Tert-Butyl Hydroquinone Assay by Surface-Enhanced Raman Scattering. Microchim. Acta.

[195] Naveen Prasad S., Anderson S.R., Joglekar M.V., Hardikar A.A., Bansal V., Ramanathan R. (2022). Bimetallic Nanozyme Mediated Urine Glucose Monitoring through Discriminant Analysis of Colorimetric Signal. Biosens. Bioelectron..

[196] Ma W., Zhang T., Li R., Niu Y., Yang X., Liu J., Xu Y., Li C.M. (2020). Bienzymatic Synergism of Vanadium Oxide Nanodots to Efficiently Eradicate Drug-Resistant Bacteria during Wound Healing In Vivo. J. Colloid. Interf. Sci..

[197] Korschelt K., Tahir M.N., Tremel W. (2018). Frontispiece: A Step into the Future: Applications of Nanoparticle Enzyme Mimics. Chem-Eur. J..

[198] Fang J., Wang H., Bao X., Ni Y., Teng Y., Liu J., Sun X., Sun Y., Li H., Zhou Y. (2020). Nanodiamond as Efficient Peroxidase Mimic against Periodontal Bacterial Infection. Carbon.

[199] Zhang H., Chhowalla M., Liu Z. (2018). 2D Nanomaterials: Graphene and Transition Metal Dichalcogenides. Chem. Soc. Rev..

[200] Zhang H., Cheng H.-M., Ye P. (2018). 2D Nanomaterials: Beyond Graphene and Transition Metal Dichalcogenides. Chem. Soc. Rev..

[201] Ruan X., Wang Y., Kwon E.Y., Wang L., Cheng N., Niu X., Ding S., Van Wie B.J., Lin Y., Du D. (2021). Nanomaterial-Enhanced 3D-Printed Sensor Platform for Simultaneous Detection of Atrazine and Acetochlor. Biosens. Bioelectron..

[202] Qiao B., Wang A., Yang X., Allard L.F., Jiang Z., Cui Y., Liu J., Li J., Zhang T. (2011). Single-Atom Catalysis of CO Oxidation Using Pt1/FeOx. Nat. Chem..

[203] Lu X., Gao S., Lin H., Yu L., Han Y., Zhu P., Bao W., Yao H., Chen Y., Shi J. (2020). Bioinspired Copper Single-Atom Catalysts for Tumor Parallel Catalytic Therapy. Adv. Mater..

[204] Lu X., Gao S., Lin H., Shi J. (2021). Single-Atom Catalysts for Nanocatalytic Tumor Therapy. Small.

[205] Wang X., Shi Q., Zha Z., Zhu D., Zheng L., Shi L., Wei X., Lian L., Wu K., Cheng L. (2021). Copper Single-Atom Catalysts with Photothermal Performance and Enhanced Nanozyme Activity for Bacteria-infected Wound Therapy. Bioact. Mater..

[206] Wang W., Luo Q., Li J., Li L., Li Y., Huo X., Du X., Li Z., Wang N. (2022). Photothermal-Amplified Single Atom Nanozyme for Biofouling Control in Seawater. Adv. Funct. Mater..

[207] Wang K., Feng D., Liu T.-F., Su J., Yuan S., Chen Y.-P., Bosch M., Zou X., Zhou H.-C. (2014). A Series of Highly Stable Mesoporous Metalloporphyrin Fe-MOFs. J. Am. Chem. Soc..

[208] Zhang Y., Sun P., Zhang L., Wang Z., Wang F., Dong K., Liu Z., Ren J., Qu X. (2019). Silver-Infused Porphyrinic Metal-Organic Framework: Surface-Adaptive, On-Demand Nanoplatform for Synergistic Bacteria Killing and Wound Disinfection. Adv. Funct. Mater..

[209] Huang S., Kou X., Shen J., Chen G., Ouyang G. (2020). “Armor-Plating” Enzymes with Metal–Organic Frameworks (MOFs). Angew. Chem. Int. Ed..

[210] Hu W., Younis M.R., Zhou Y., Wang C., Xia X. (2020). In Situ Fabrication of Ultrasmall Gold Nanoparticles/2D MOFs Hybrid as Nanozyme for Antibacterial Therapy. Small.

[211] Zhao X., He X., Hou A., Cheng C., Wang X., Yue Y., Wu Z., Wu H., Liu B., Li H. (2022). Growth of Cu _2_ O Nanoparticles on Two-Dimensional Zr–Ferrocene–Metal–Organic Framework Nanosheets for Photothermally Enhanced Chemodynamic Antibacterial Therapy. Inorg. Chem..

[212] Carrillo-Carrión C., Comaills V., Visiga A.M., Gauthier B.R., Khiar N. (2023). Enzyme-Responsive Zr-Based Metal–Organic Frameworks for Controlled Drug Delivery: Taking Advantage of Clickable PEG-Phosphate Ligands. ACS Appl. Mater. Inter..

[213] Shen M., Liao X., Xianyu Y., Liu D., Ding T. (2022). Polydimethylsiloxane Membranes Incorporating Metal–Organic Frameworks for the Sustained Release of Antibacterial Agents. ACS Appl. Mater. Interfaces.

